# A General Access Route to High‐Nuclearity, Metal‐Functionalized Molecular Vanadium Oxides

**DOI:** 10.1002/anie.202114548

**Published:** 2022-01-17

**Authors:** Simon Greiner, Jan Hettig, Alec Laws, Katharina Baumgärtner, Jenna Bustos, Ann‐Christin Pöppler, Adam H. Clark, May Nyman, Carsten Streb, Montaha Anjass

**Affiliations:** ^1^ Institute of Inorganic Chemistry I Ulm University Albert-Einstein-Allee 11 89081 Ulm Germany; ^2^ Helmholtz Institute Ulm (HIU) Helmholtzstraße 11 89081 Ulm Germany; ^3^ Department of Chemistry Oregon State University Corvallis OR 97331 USA; ^4^ Institute of Organic Chemistry University of Wuerzburg Am Hubland 97074 Wuerzburg Germany; ^5^ Paul Scherrer Institute Forschungsstraße 111 5232 Villingen Switzerland

**Keywords:** Polyoxometalate, Self-assembly, Supramolecular, Synthesis, Vanadium oxide

## Abstract

Molecular metal oxides are key materials in diverse fields like energy storage and conversion, molecular magnetism and as model systems for solid‐state metal oxides. To improve their performance and increase the variety of accessible motifs, new synthetic approaches are necessary. Herein, we report a universal, new precursor to access different metal‐functionalized polyoxovanadate (POV) clusters. The precursor is synthesized by a novel solid‐state thermal treatment procedure. Solution‐phase test reactions at room temperature and pressure show that reaction of the precursor with various metal nitrate salts gives access to a range of metal‐functionalized POVs. The first nitrate‐templated molecular calcium vanadate cluster is reported. We show that this precursor could open new access routes to POV components for molecular magnetism, energy technologies or catalysis.

## Graphical abstract

Controlled thermal treatment of the common decavanadate cluster in the solid state yields a universal precursor for the synthesis of large, metal‐functionalized polyoxovanadates, as demonstrated for three examples.

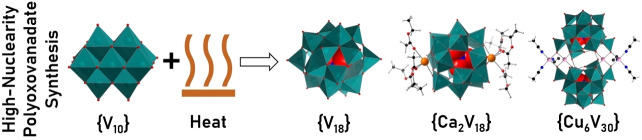

## Introduction

Owing to their wide structural and chemical variety, metal oxides are key materials for many modern technologies.^[1]^ However there remain major challenges in their controlled and predictable synthesis, as well as targeted modification and functionalization. In this respect, molecular metal oxides, so‐called polyoxometalates (POMs), are important model systems which enable rational materials design and provide structure–property–function correlations on the molecular level.[^2^, ^3^] POMs are anionic metal oxide clusters of the early transition metals (often W, Mo and V) in their high oxidation states, formed by self‐assembly in solution.^[4]^ Due to their well‐defined structures, they can be leveraged as molecular analogues of solid‐state metal oxides.^[5]^ Additionally, their versatile electrochemistry and controlled tuneability make POMs valuable materials for energy conversion and storage,[^6^, ^7^] (photo‐)catalysis[^8^, ^9^] as well as molecular electronics and spintronics.[^10^, ^11^]

While initial work has mainly been focused on molybdenum‐ and tungsten‐based clusters, polyoxovanadates (POVs) have gained more attention in the past decades.[^12^, ^13^, ^14^] This can be explained by the increased structural flexibility of vanadium, which leads to higher synthetic complexity.[^10^, ^15^] At the same time, the unique electronic structure, rich electrochemistry and coordinative flexibility make polyoxovanadates ideal components for (post‐)lithium batteries,[^16^, ^17^] (non‐aqueous) redox‐flow batteries,[^18^, ^19^] molecular magnetism[^20^, ^21^] and (oxidation‐) catalysis.[^22^, ^23^, ^24^] Recent studies have therefore concentrated on the structural[^15^, ^25^, ^26^] and electrochemical[^27^, ^28^, ^29^] tuning of POVs. To‐date, the targeted synthesis of new, metal‐functionalized POVs remains challenging. However, this ability is of fundamental importance, as structural and chemical modification are keys for POV reactivity tuning.^[30]^

One main challenge in POV synthesis is the high chemical stability of the decavanadate anion [H_*x*_V_10_O_28_]^(6−*x*)^ under commonly used (aqueous) conditions.^[31]^ In contrast, POV synthesis in organic solvents has recently attracted widespread attention and offers vast possibilities to access new cluster structures and therefore, new functions.[^30^, ^32^] Complex assembly mechanisms and the limited number of organo‐soluble precursors make targeted materials development difficult.^[13]^ Thus, access to highly reactive precursors for controlled POV formation is essential to enable fast‐paced progress in the field.

Recent studies have demonstrated that “redox‐activation”, i.e. the reduction and re‐oxidation of POV precursors is a useful approach to generate reactive vanadate precursors and intermediates.[^33^, ^34^] Systematic investigation showed, that the presence of mixed‐valent species or oxidation‐driven synthesis starting from reduced clusters are promising approaches.^[35]^ Recent examples include the synthesis of (*n*Bu_4_N)_4_[Cu_6_(CH_3_CN)_6_V_30_O_82_(NO_3_)_2_] (=**{Cu_6_V_30_}**) under oxidative conditions from fully reduced [H_6_V^IV^_18_O_42_]^6−^,^[34]^ and the synthesis of a [V_16_O_38_Br]^6−^ from [V^IV^_2_V^V^_8_O_26_]^4−^ in the presence of *p*‐toluenesulfonic acid and (*n*Bu_4_N)Br in MeCN.^[36]^ For the latter, the synthesis of a chloride‐templated analogue required additional reducing agent due to the less‐negative reduction potential of chloride compared with bromide. In a related study, the assembly of the oxygen‐deficient Lindqvist‐type POV‐alkoxide cluster [V_6_O_6_(OC_2_H_5_)_12_] starting from [VO(OEt)_3_] was investigated and an intermediate cyclic [VO(OEt)_2_]_6_ species was isolated.^[37]^ By isolation of the intermediate, the contribution of reducing agents on the assembly process could be demonstrated.

Building on these ground‐breaking studies, we report synthetic access to a highly reactive yet benchtop‐stable vanadate precursor. The compound is characterized in the solid state and in solution, and its versatile reactivity is exemplified by accessing three high‐nuclearity POV clusters. POV researchers can now utilize this approach to access new POV‐clusters and derivatives.

## Results and Discussion

The title compound **1** is synthesized by careful thermal treatment of the stable decavanadate cluster (*n*Bu_4_N)_3_[H_3_V_10_O_28_] (=**{V_10_}**). Starting at ambient conditions (room temperature, air atmosphere), the sample was heated to 195 °C (heating rate: 200 °C h^−1^). The sample was kept at 195 °C for 2 h, and then cooled to room temperature. This resulted in quantitative conversion to a dark brown powder, which is soluble (without residue) in acetonitrile. This result is surprising, as it was recently reported by some of us, that thermal treatment of the decavanadate Li_6_[V_10_O_28_] under vacuum results in the formation of solid‐state oxides (specifically LiV_3_O_8_ and LiVO_3_).^[38]^ We speculate that the bulky *n*Bu_4_N^+^ organo‐cations enable a different reaction path, possibly by separating individual decavanadate clusters sufficiently to enable conversion without solid‐state oxide formation.

**Figure 1:**
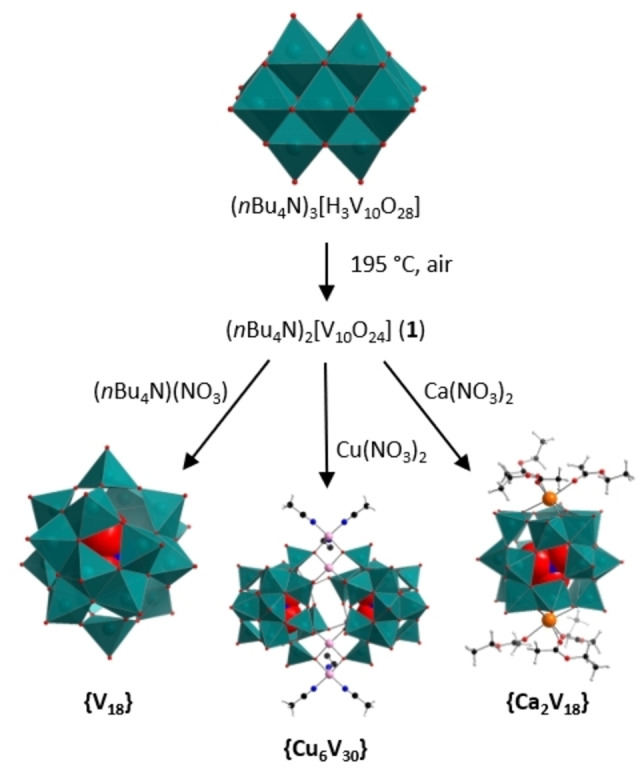
Reaction scheme showing the different motifs obtained by reaction of **1** with different nitrate salts. Color scheme: V atoms and [VO_5_] polyhedra: teal, Cu: pink, Ca: orange, O: red, N: blue, C: black, H: grey.

Combined elemental analysis (ICP‐OES and CHN analysis, see Supporting Information) and thermogravimetric analysis (TGA, see Supporting Information, Figure S6) allowed us to propose the sum formula (*n*Bu_4_N)_2_[V_10_O_24_] (=**1**) (Figure 1). Notably, these data suggest, that the initial thermal treatment of **{V_10_}** results in the selective oxidative loss of one *n*Bu_4_N^+^ cation per cluster formula unit, accompanied by the formal loss of four oxygen atoms per decavanadate. Further, infrared‐ and ^1^H NMR ‐spectroscopies of **1** show the presence and structural integrity of the two remaining *n*Bu_4_N^+^ cations, see Supporting Information. One interpretation would be that the selective oxidation of one (*n*Bu_4_N)^+^ under the conditions reported occurs via a Mars–van–Krevelen mechanism—i.e. the loss of POM‐based surface oxygens—has been reported previously for vanadium‐containing POMs.[^39^, ^40^, ^41^] When **1** is further heated to ca. 330 °C, TGA indicates the loss of the two remaining *n*Bu_4_N^+^ cations (obs.: 36.0 wt. %; calc.: 35.2 wt. %, Supporting Information, Figure S6). Note that heating of **{V_10_}** for longer periods and higher temperatures than used in the synthesis of **1** lead to insoluble products, which were not investigated further.

X‐ray photoelectron spectroscopy (XPS) of **1** showed a pronounced shoulder in the V 2p_3/2_ region, indicating a mixed valent species, suggesting that thermal treatment **{V_10_}** of the precursor is accompanied by partial V^V/IV^ reduction. This is in line with charge‐balance considerations, which suggest an atomic ratio of V^IV^ : V^V^=4 : 6 in **1**, resulting in the formula (*n*Bu_4_N)_2_[V^IV^_4_V^V^_6_O_24_]. Fitting of the deconvoluted vanadium XPS spectrum indicates an atomic ratio of V^IV^ : V^V^ of 3.2 : 6.8 (see Supporting Information, Figure S5).^[42]^ The difference observed might be related to surface oxidation effects, deviations due to the complex fitting process.^[43]^

Note that the formula reported for **1** is purely a sum formula based on the data obtained, it does not imply any structural suggestions as to the structure of the vanadate species present. At the same time, a one‐oxygen deficient decavanadate cluster [(*n*Bu_4_N)_2_[HV^V^_10_O_27_]^−^ has been observed as a fragment during ESI‐MS by Cronin and co‐workers, suggesting that oxygen loss is principally possible for decavanadates.^[44]^ Additionally, the loss of terminal oxygen atoms in reduced POV clusters has also recently been reported for Lindqvist‐type POV‐alkoxides by Matson and colleagues.[^24^, ^40^]

**Figure 2:**
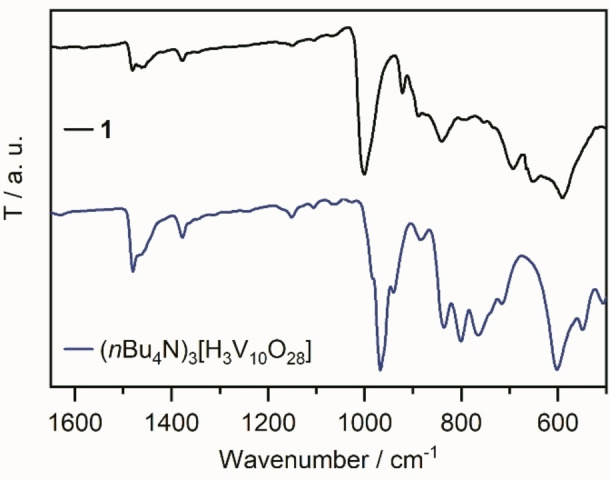
Infrared spectra of **1** (black) and the precursor **{V_10_}** (blue) in the characteristic range between 1650–500 cm^−1^.

Comparative infrared spectroscopy of **1** and the precursor **{V_10_}** indicates significant structural changes during the conversion: the ν(V=O_terminal_) mode shifts from 968 cm^−1^ in **{V_10_}** to 997 cm^−1^ in **1**, indicating an increasing bond‐strength in **1**. At the same time, the ν(V−O−V) bands at 770 cm^−1^ and 803 cm^−1^ vanish, while the ν(V−O−V) bands at 840 cm^−1^ and 888 cm^−1^ stay virtually unchanged. This data suggests distinct structural changes of the original vanadium oxide cluster framework. Note, that the bands associated with the *n*Bu_4_N^+^ (between 1380 and 1480 cm^−1^ in Figure 2) remain spectrally virtually unchanged but feature lower intensity. This is expected as during the conversion of **{V_10_}** to **1**, one *n*Bu_4_N^+^ is lost.

**Figure 3:**
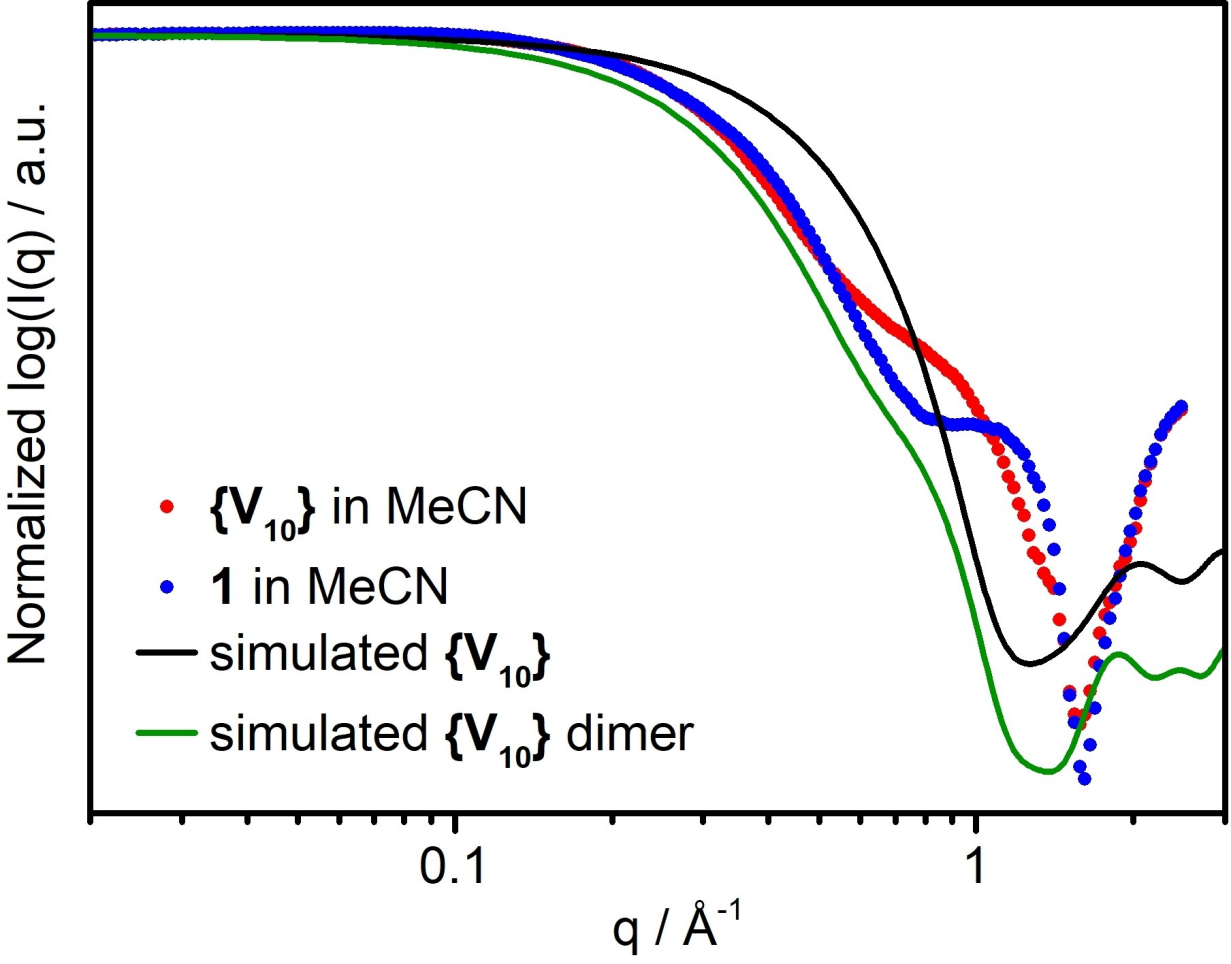
Experimental and simulated scattering curves, intensity normalized to *I*(*q*)_max_ for ease of comparison. Experimental scattering curves are from 100 mM solutions in acetonitrile.

Attempts to recrystallize **1** to obtain single crystals for X‐ray diffraction remained unsuccessful. Thus, we performed extensive solution studies to gain insights into chemical changes and reactivity of **1** upon dissolution. First, acetonitrile solutions of **1** were studied by small‐angle X‐ray diffraction (SAXS) at four different concentrations (10 mM, 25 mM, 50 mM and 100 mM). As a comparison, identical experiments were performed using the precursor **{V_10_}**, see Supporting Information, Figure S3. Both systems show low dispersity with minimal aggregation (indicated by the plateau region at *q*<0.1 Å^−1^), and no changes in speciation with variable concentration, as indicated by minimal differences between the scattering curves (other than intensity). Comparison of the SAXS data of the most concentrated solutions (100 mM, see Figure 3) highlights the similarity in the Guinier region below *q*=0.45 Å^−1^, suggesting a similar size and shape of the dominant scattering species for **1** and **{V_10_}**. In earlier studies of **{V_10_}** in non‐polar solvents, the main scattering species has been identified as H‐bonded dimer.^[45]^ Comparison of our experimental scattering to simulated scattering of isolated **{V_10_}** clusters and **{V_10_}** dimers indicates that the dimer is also the dominant species in our **{V_10_}** solutions. However, clear differences between **1** and **{V_10_}** at *q*>0.45 Å^−1^ are observed. Since the differences are robustly preserved across the studied concentration range, this represents a true difference between **1** and **{V_10_}** solutions and cannot be completely attributed to counter‐cation or solvation effects. The radii of gyration (*R*_g_, a shape‐independent assessment of size of scattering species) determined^[46]^ for the 100 mM solutions are 4.38 Å (**1**) and 4.56 Å (**{V_10_}**), the slightly smaller size for **1** being consistent with loss of oxo‐ligands. Note, however, that the solid, as‐synthesized **1**, and 1 dissolved in MeCN are not identical species (see below), so that only qualitative comparison of the observed trends is possible. Further experiments and theoretical calculations are required for conclusive description of solution speciation of **1**.

Dissolving **1** in acetonitrile and immediate recovery by solvent evaporation (see Supporting Information section 5 for details) indicates spontaneous reactivity of **1** in solution: comparison of the characteristic infrared spectra of as‐prepared **1** and the recovered sample shows distinct differences in the V−O‐fingerprint region, highlighting that **1** undergoes structural changes under the reaction conditions described (see Supporting Information, Figure S2).

Further investigation of the solution speciation of **1** in acetonitrile (ca. 5×10^−5^ M) by high‐resolution electrospray ionization mass‐spectrometry (HR‐ESI‐MS) supports this reactivity. Analysis of the data using *m*/*z* and isotopic pattern assignments showed a plethora of different species (see Supporting Information, Table S5 and Figure S13). Analysis of the HR‐ESI‐MS at high *m*/*z* values showed many high‐nuclearity ({V_5_}–{V_36_}), mixed‐valent vanadium oxide species. For example, the most intense signal can be assigned to (*n*Bu_4_N)_3_[H_3_V^IV^_8_V^V^_10_O_52_]^2−^ (obs.: *m*/*z*=1239.29 calc.: *m*/*z*=1239.30). Other large vanadium oxide species observed include [V^IV^_32_V^V^_4_O_74_]^2−^ (obs.: *m*/*z*=1508.84, calc.: *m*/*z*=1508.80) and [H_3_V^IV^_11_V^V^_17_O_67_]^2−^ (obs.: *m*/*z*=1250.55, calc.: *m*/*z*=1250.55). To the best of our knowledge, no vanadates with these stoichiometric compositions have been reported to‐date, but the V : O ratios are within the range expected for polyoxovanadates. These species could therefore be new clusters or intermediates, formed from **1** upon solution‐ or gas‐phase speciation under ESI‐MS conditions. Future work will aim at exploring and stabilizing these species, e.g. by using bulky organo‐cations^[47]^ or stabilizing external ligands (e.g. phosphonates or arsonates).[^48^, ^49^, ^50^] Additionally, the known mixed‐valent cluster (*n*Bu_4_N)_2_[V^IV^_2_V^V^_8_O_26_]^2−^ can be observed *m*/*z*=1409.90 (calc.: *m*/*z*=1409.88).^[51]^

In essence, this leads us to suggest that upon dissolution in acetonitrile, **1** easily forms reactive oxo‐vanadium species which then undergo cluster self‐assembly. This in turn suggests that the resulting vanadate structures are dependent on the assembly conditions, e.g. solvent, presence of template anions, presence of metal cations, *etc*. This suggests that **1** can be proposed as a “universal”, highly reactive precursor for the assembly of novel polyoxovanadate species.

**Figure 4:**
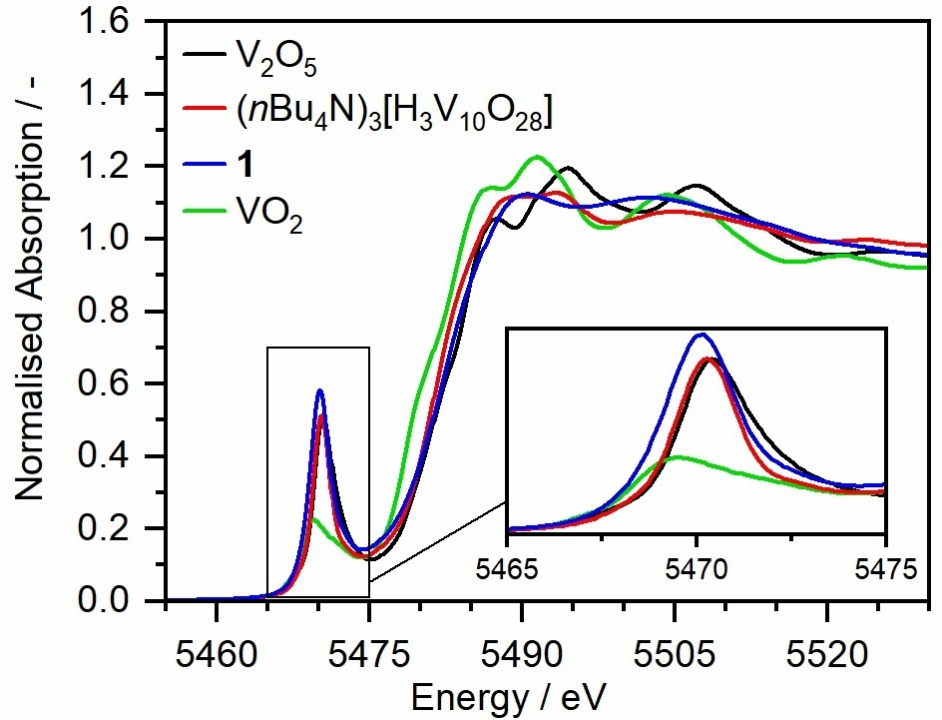
V K‐edge XANES data comparing **{V_10_}** and **1** with VO_2_ and V_2_O_5_ references.

To gain further chemical and structural information on **1**, X‐ray absorption fine‐structure spectroscopy (XAFS) was performed at the SuperXAS beamline of the Swiss Light Source SLS (details see Supporting Information). Near‐edge analysis (XANES) of the vanadium K‐edge suggests high structural similarity between **1** and **{V_10_}**, based on the resemblance between their respective near‐edge spectra (see Figure 4). Additionally, a shift of the pre‐edge feature to slightly lower energies is observed. This could be a result of change in local geometry, e.g. from octahedral to square‐pyramidal (of some V atoms), or it could indicate partial V^V/IV^ reduction as indicated by the XPS results (see above).[^52^, ^53^]

Analysis of the extended X‐ray absorption fine‐structure (EXAFS) spectra shows some distinct changes while the overall structure seems similar to the initial decavanadate cluster (see Supporting Information, section 9b). The vanadium atoms seem to remain octahedrally coordinated, although the longest V−O interactions (V−O_4_, see Supporting Information, Table S3 and S4) present for the decavanadate is absent in **1**. This could be either caused by shortening of the respective bond (and distribution of the contribution over the remaining V−O interactions, as modelled here), increased disorder or the loss of these oxygen atoms. Due to the complex structure of the cluster and averaging over several V centers, it is not possible to make a definitive attribution. Additionally, the magnitude of the V−V interaction is noted to decrease significantly. Again, this could be caused by increased disorder as well as (partial) fragmentation of the cluster. At the same time, the contribution does not vanish completely, indicating that the compound remains in a cluster structure and does not decompose to mononuclear vanadium complexes. However, no definite structure can be derived from the data.

Further insight into the structure of **1** in the solid state was obtained by magic angle spinning (MAS)‐^51^V NMR of **1** and **{V_10_}. {V_10_}** showed three signals at −440 ppm, −505 ppm and −521 ppm, which agree with the signals and chemical shifts expected for the decavanadate, based on solution data.^[54]^ A fourth signal, which is expected from solution measurements, is hidden underneath a strong spinning sideband (see Supporting Information, Figure S10). In line with solution data, the intensities for signals <−500 ppm are also stronger than for signal >−500 ppm.

For **1**, broader and less intense signals are observed at −492 ppm, −520 to −540 ppm, and −562 ppm and remain at similar shifts as the pristine decavanadate (see Supporting Information, Figure S11). Additionally, a very dominant, sharp signal is observed at −628 ppm. Based on the lack of spinning sidebands and small line width of this signal, it is expected to be either very symmetric or exhibit high positional averaging. It is important to emphasize that the postulated V^IV^ sites are typically undetectable by NMR spectroscopy due to the presence of an unpaired electron per V center.^[55]^ However, the shift of the broad signals to lower ppm values is expected to be due to the presence of reduced V^IV^ centers in **1**. This is supported by ^51^V NMR experiments on two known dodecavanadate species,[^26^, ^27^] where a shift to lower ppm values is observed for the cluster with 11 V^V^ and 1 V^IV^ environment^[27]^ as compared to the fully oxidized compound (see Supporting Information section 10).^[26]^ Furthermore, the distribution of intensities is different from the **{V_10_}** starting material.

The data in general indicates only a limited number of species in **1**, possibly even a single molecular structure. First indications hint at some similarity to the decavanadate cluster, but further investigations are necessary to unambiguously determine the structure of this compound.

Based on these results, we proposed, that **1** might be a highly reactive precursor for the formation of novel POVs. First, as initial proof of principle, and to explore the template‐induced formation of POVs, we chose nitrate as anionic template, which is well‐known to form high‐nuclearity POVs.[^56^, ^57^, ^58^] To this end, **1** was mixed with (*n*Bu_4_N)(NO_3_) in acetonitrile and stirred overnight at room temperature. Addition of ethyl acetate (EtOAc) into the reaction mixture gave dark brown single crystals of the literature‐known nitrate‐templated, chiral POV (*n*Bu_4_N)_5_[V_18_O_46_(NO_3_)] (=**{V_18_}**, see Figure 1) in yields of 69 % based on V. The species was identified by single‐crystal X‐ray diffraction (Supporting Information, Table S2),[^58^, ^59^] XPS, UV/Vis‐ and IR spectroscopy as well as ICP‐OES (see Supporting Information).

**{V_18_}** has only been isolated and reported once by Hayashi and co‐workers,^[58]^ who obtained it by reaction of (*n*Bu_4_N)_4_[V^IV^_2_V^V^_8_O_26_] with nitric acid and *tert*‐butyl hydroperoxide in nitroethane. In contrast to these highly oxidative conditions, the synthesis reported here can be performed under mild, room‐temperature conditions.

Cyclic voltammetry of **{V_18_}** in acetonitrile shows five (quasi‐)reversible redox processes at I/I′=0.67 V, II/II′=0.33 V, III/III′=−0.43 V, IV/IV′=−0.79 V and V/V′=−1.34 V (all vs. Fc^+^/Fc, see Supporting Information, Figure S12). Note, that Hayashi and co‐workers only reported the two waves I/I′ and II/II′ in oxidative direction and showed that both are reversible one‐electron processes resulting in a fully oxidized cluster. However, the three remaining waves (all in reductive direction) have not been described yet and suggest, that **{V_18_}** might be an interesting example for multiple (proton‐coupled) electron transfer, e.g. for electrochemical energy storage applications.^[30]^

Next, we investigated the reaction of **1** with copper(II) ions, as earlier results showed the formation of one of the largest POV species from mixed‐valent precursors with copper.^[34]^ Therefore, we reacted **1** with Cu(NO_3_)_2_ ⋅ 3 H_2_O in acetonitrile (details see Supporting Information section 3). The reaction gave dark brown single crystals of the literature‐known copper‐vanadate cluster (*n*Bu_4_N)_4_[Cu_6_(CH_3_CN)_6_V_30_O_82_(NO_3_)_2_] (=**{Cu_6_V_30_}**, see Figure 1) in yields of 43 % based on V. The sample identify was confirmed by single‐crystal X‐ray diffraction (Supporting Information, Table S2),[^34^, ^59^] infrared spectroscopy and ICP‐OES (see Supporting Information). Note that all vanadium atoms in **{Cu_6_V_30_}** are V^V^, while previous studies suggest that the presence of some V^IV^ species is required to initiate cluster assembly.^[34]^ Also, note that **{Cu_6_V_30_}** can be accessed from fully reduced [H_6_V_18_O_42_]^6−^ presumably via a the copper‐catalyzed oxidation under ambient atmosphere. Note, that both **{V_18_}** and **{Cu_6_V_30_}** exhibit pentagonal building units related to the [M_6_O_21_] units (M=Mo, W) observed in large molybdenum‐ and tungsten‐based polyoxometalate clusters.[^60^, ^61^] They are speculated to play a crucial role in the formation and growth process.[^3^, ^62^]

**Figure 5:**
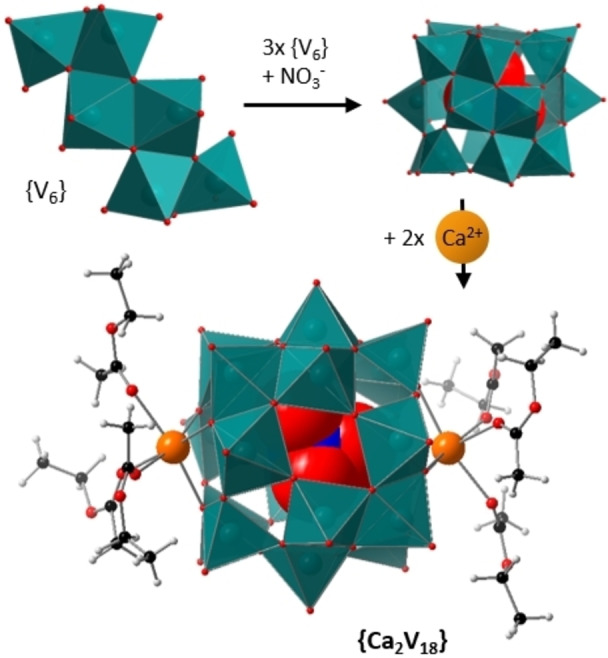
Building blocks and structure of **{Ca_2_V_18_}**: Three {V_6_} zigzag units are wrapped around a central nitrate template. Each {V_6_} unit is connected to both other {V_6_} units by four corner‐shared oxygen atoms. On both ends, a trigonal binding site is occupied by Ca^2+^‐ions. The coordination sphere of the calcium ions is completed by three ethyl acetate molecules, leading to an octahedral coordination environment. Color scheme: V atoms and [VO_5_] polyhedral: teal, Ca: orange, O: red, N: blue, C: black, H: grey.

As a third test reaction, we decided to build on the earlier reactivity studies, see above, and opted to explore an unknown reaction system, i.e. nitrate‐templated calcium vanadate clusters. This concept is based on recent results by some of us, where alkaline earth metal‐functionalized POVs showed surprising structural and electrochemical effects.[^27^, ^63^] To this end, **1** was reacted with Ca(NO_3_)_2_ ⋅ 4 H_2_O in acetonitrile and stirred overnight (Details see Supporting Information section 3). Addition of ethyl acetate into the reaction mixture gave dark yellow crystals (yield: 27 % based on V). Single‐crystal X‐ray diffraction gave the formula (*n*Bu_4_N)_3_[Ca_2_(EtOAc)_6_V_18_O_48_(NO_3_)] (**{Ca_2_V_18_}**) (Figure 5).^[59]^ **{Ca_2_V_18_}** crystallizes in the monoclinic space group *P*2_1_/*c* with cell axes *a*=27.641 Å, *b*=16.3250 Å and *c*=28.068 Å, and angles *α*=*γ*=90° and *β*=109.915°, *V*=6296.0 Å^3^ (for crystallographic details see Supporting Information, Table S1). Compound identity and purity were further confirmed by elemental analysis (CHN, ICP‐OES, thermal analysis (TGA), see Supporting Information.

The cluster is assembled from three isostructural zigzag {V_6_} units. Each {V_6_} unit is formed by three dinuclear, edge‐sharing [VO_5_] square pyramids, which are then linked to the neighboring dinuclear species. V−O bond lengths are all within the expected range (*d*_V=O(terminal)_)=1.59–1.60 Å, *d*_V−O(bridging)_=1.74–1.98 Å). Each {V_6_} unit is connected to neighboring {V_6_} groups by four shared oxo corners, forming an almost circular barrel around the central nitrate template. In contrast to **{V_18_}**, the planar structure of the template is not reflected in this cluster framework. Three μ^2^‐oxo ligands are located on the top and at the bottom of the barrel‐shaped cluster giving rise to one binding sites on each side of the cluster. In **{Ca_2_V_18_}** these binding sites are occupied by Ca^2+^ ions (*d*_Ca−O_=2.32–2.34 Å). The coordination sphere of Ca^2+^ is completed by three EtOAc ligands (*d*_Ca−O(EtOAc)_)=2.36–2.38 Å), leading to an octahedral coordination environment. Additionally, long‐range electrostatic interactions between the Ca^2+^‐ions and the central nitrate template (*d*_Ca−O(Nitrate)_=3.31–3.39 Å) are observed. Notably, the {V_6_} groups in **{Ca_2_V_18_}** introduce helical chirality into the cluster, although no chirality is present in the starting material. A similar effect has been observed in the case of **{V_18_}**, but remains scarce in polyoxovanadates.[^58^, ^64^] Note that the crystalline compound is a racemic mixture of both enantiomers.

HR‐ESI‐MS measurements of **{Ca_2_V_18_}** in acetonitrile (ca. 5×10^−5^ M) indicates, that the cluster can be transferred into the gas‐phase (see Supporting Information, Table S6 and Figure S14). The full cluster {(*n*Bu_4_N)[Ca_2_V^V^_18_O_48_(NO_3_)]}^2−^ is detected as most intense signal at *m*/*z*=1034.48 (calc.: *m*/*z*=1034.47). Also, species with more than one cluster unit can be observed e.g. {(*n*Bu_4_N)_3_[Ca_2_V^V^_18_O_48_(NO_3_)]_2_}^3−^ at *m*/*z*=1460.08 (calc.: *m*/*z*=1460.06). Similar observations have recently been reported by some of us^[27]^ for a di‐calcium functionalized dodecavanadate and were assigned to the formation of oligomeric chains. However, in contrast to these earlier studies, the fragments observed here do not contain bridging solvent molecules in the crystal lattice. While the coordinating ethyl acetate molecules could be stripped off during the ESI‐MS measurement, future investigations will clarify whether oligomeric species are present in solution or can be achieved by introducing appropriate solvents or bridging ligands.^[63]^ Furthermore, the anion {H_2_[Ca_2_V^IV^_9_V^V^_9_O_44_(NO_3_)]}^2−^ is observed at *m*/*z*=882.34 (calc.: *m*/*z*=882.34). Note, that a similar mixed‐valent framework, [HV^IV^_12_V^V^_6_O_44_(NO_3_)]^10−^, has been reported by Müller and colleagues.^[65]^ We assigned this observation to a cluster degradation, indicating a low stability of the cluster in solution. This is supported by the UV/Vis spectroscopic observation of characteristic intervalence charge‐transfer (IVCT) bands and a characteristic shoulder in the XPS spectrum, indicating the presence of electronically coupled V^IV/V^ species However, note that ESI‐MS, charge balance considerations and bond valence sum calculations suggest all vanadium atoms in **{Ca_2_V_18_}** are fully oxidized.

**Figure 6:**
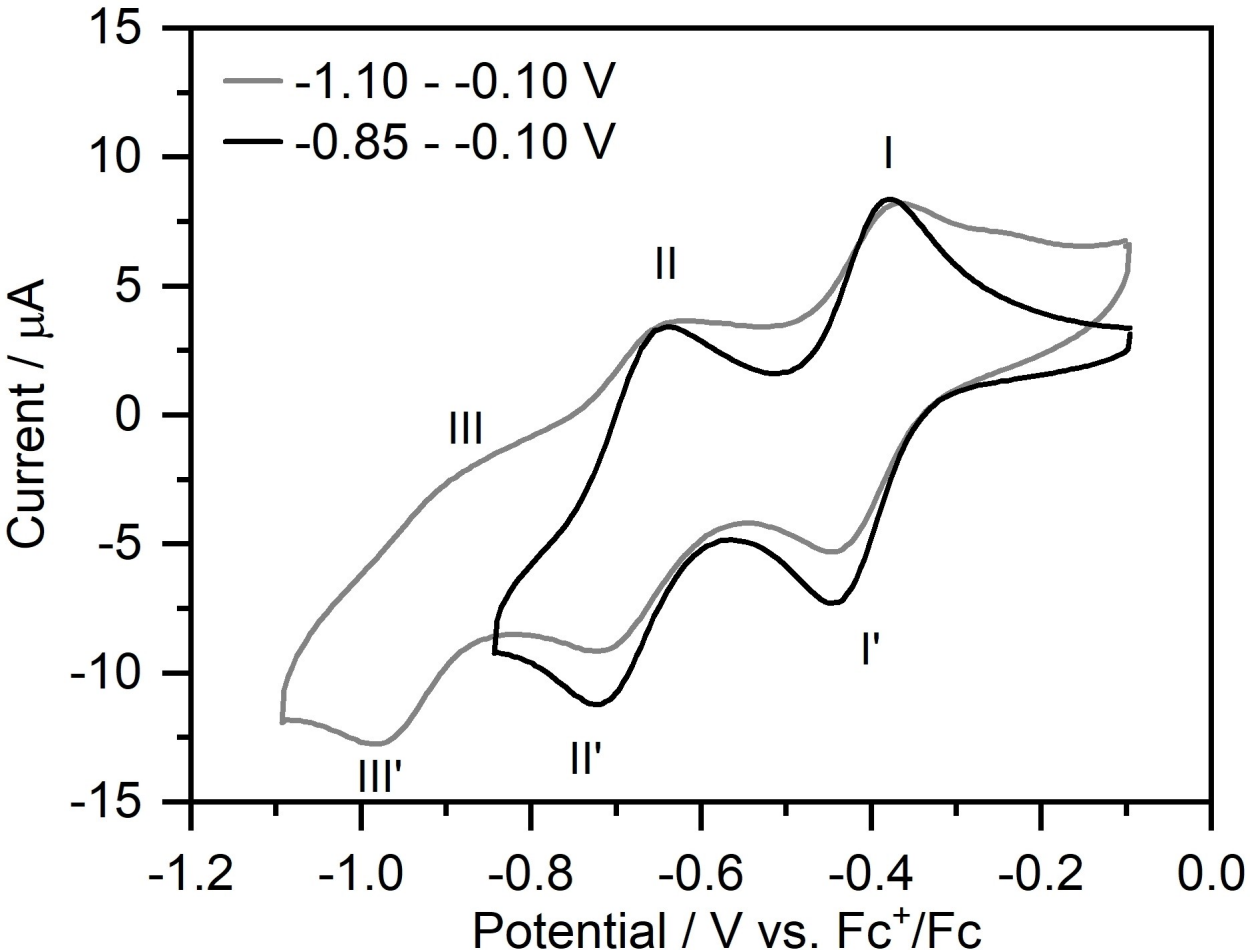
Cyclic voltammogram of **{Ca_2_V_18_}** (1 mM) in a potential range between −0.85–−0.10 V (black) and −1.10–−0.10 V (grey). Conditions: anhydrous, de‐oxygenated DMF (0.1 M (*n*Bu_4_N)PF_6_), scan rate: 0.1 V s^−1^.

This is further supported by cyclic voltammetry of freshly synthesized **{Ca_2_V_18_}** in *N,N‐*dimethyl‐formamide (DMF). Two (quasi‐)reversible redox processes are observed in cathodic direction of the open‐circuit potential (OCP; *E*=−0.36 V) at I/I′=−0.41 V and II/II′=−0.68 V vs. Fc^+^/Fc (see Figure 6). Note the proximity of the OCP to the first reduction, suggesting facile reducibility under ambient conditions. When comparing the electrochemical activity of **{Ca_2_V_18_}** with the recently reported calcium vanadate (*n*Bu_4_N)_2_[Ca_2_(DMF)_3_V_12_O_32_Cl] (=**{Ca_2_V_12_}**), we note that **{Ca_2_V_12_}** shows five (quasi‐)reversible processes.^[27]^ In contrast, for **{Ca_2_V_18_}**, we observe irreversible reduction processes at potentials lower *E*=−0.85 V (Supporting Information, Figure S12). We propose that this might be linked to the high negative charge of **{Ca_2_V_18_}** (5−), so that stabilization, e.g. by cation association with protons or others might be a suitable route to enhance electron storage capacity.^[66]^

## Conclusion

In conclusion, we herein report a universal precursor for large polyoxovanadate frameworks. In three test reactions, we demonstrated the versatility by simple stirring of this “magic” compound with different metal nitrate salts leading to entirely different clusters. This new synthetic route can be leveraged to synthesize novel polyoxovanadate clusters with promising properties for energy storage/conversion applications. Future works will explore this new synthetic approach further, and will also study the properties of the mixed‐valent vanadate precursor, e.g. with respect to sensing and energy storage.

## Conflict of interest

There are no conflicts to declare.

1

## Supporting information

As a service to our authors and readers, this journal provides supporting information supplied by the authors. Such materials are peer reviewed and may be re‐organized for online delivery, but are not copy‐edited or typeset. Technical support issues arising from supporting information (other than missing files) should be addressed to the authors.

Supporting Information

Supporting Information

Supporting Information

Supporting Information

Supporting Information

Supporting Information

Supporting Information

## Data Availability

The data that support the findings of this study are available from the corresponding author upon reasonable request.
