# Supplementary material for: A General Access Route to High‐Nuclearity, Metal‐Functionalized Molecular Vanadium Oxides
Source: Angew Chem Int Ed Engl. 2022 Jan 17;61(9):e202114548. doi: 10.1002/anie.202114548 (PMC9302674; doi:10.1002/anie.202114548)

checkCIF/PLATON report

Structure factors have been supplied for datablock(s) sg20090_0m

THIS REPORT IS FOR GUIDANCE ONLY. IF USED AS PART OF A REVIEW PROCEDURE FOR PUBLICATION, IT SHOULD NOT REPLACE THE EXPERTISE OF AN EXPERIENCED CRYSTALLOGRAPHIC REFEREE.

No syntax errors found. CIF dictionary Interpreting this report

Datablock: sg20090_0m

```
Bond precision:  C-C = 0.0146 A          Wavelength=0.71073

Cell:            a=27.641(2)     b=16.3250(14)       c=28.068(2)
                 alpha=90        beta=105.915(2)     gamma=90
Temperature:     150 K
```

	Calculated	Reported
Volume	12179.9(16)	12179.9(17)
Space group	P 21/c	P 1 21/c 1
Hall group	-P 2ybc	-P 2ybc
Moiety formula	C24 H48 Ca2 O60 V18, 2(C16 H36 N), C14 H31 N, N O3, C2 H5	C24 H48 Ca2 O60 V18, 3(C16 H36 N), N O3
Sum formula	C72 H156 Ca2 N4 O63 V18	C72 H156 Ca2 N4 O63 V18
Mr	3083.09	3083.08
Dx,g cm-3	1.681	1.681
Z	4	4
Mu (mm-1)	1.485	1.485
F000	6296.0	6296.0
F000’	6322.49	
h,k,lmax	34,20,35	34,20,35
Nref	25862	25799
Tmin,Tmax	0.659,0.729	0.649,0.731
Tmin’	0.505	

```
Correction method= # Reported T Limits: Tmin=0.649 Tmax=0.731
AbsCorr = MULTI-SCAN

Data completeness= 0.998          Theta(max)= 26.733

R(reflections)= 0.0740( 19459)    wR2(reflections)= 0.2071( 25799)

S = 1.112                   Npar= 1453
```

The following ALERTS were generated. Each ALERT has the format
test-name_ALERT_alert-type_alert-level.
Click on the hyperlinks for more details of the test.

Alert level A

```
PLAT910_ALERT_3_A Missing # of FCF Reflection(s) Below Theta(Min).         62 Note
```

Alert level B

```
PLAT413_ALERT_2_B Short Inter XH3 .. XHn     H35A     ..H71B     .       1.92 Ang.
                                                    x,y,z  =     1_555 Check
PLAT430_ALERT_2_B Short Inter D...A Contact  O1       ..O62      .       2.73 Ang.
                                                    x,y,z  =     1_555 Check
PLAT430_ALERT_2_B Short Inter D...A Contact  O2       ..O61      .       2.73 Ang.
                                                    x,y,z  =     1_555 Check
PLAT430_ALERT_2_B Short Inter D...A Contact  O5       ..O65      .       2.79 Ang.
                                                    x,y,z  =     1_555 Check
PLAT430_ALERT_2_B Short Inter D...A Contact  O14      ..O62      .       2.84 Ang.
                                                    x,y,z  =     1_555 Check
PLAT430_ALERT_2_B Short Inter D...A Contact  O16      ..O64      .       2.80 Ang.
                                                    x,y,z  =     1_555 Check
```

Alert level C

```
DIFMX02_ALERT_1_C  The maximum difference density is > 0.1*ZMAX*0.75
            The relevant atom site should be identified.
RINTA01_ALERT_3_C  The value of Rint is greater than 0.12
            Rint given   0.128
PLAT020_ALERT_3_C The Value of Rint is Greater Than 0.12 .........      0.128 Report
PLAT094_ALERT_2_C Ratio of Maximum / Minimum Residual Density ....       2.33 Report
PLAT097_ALERT_2_C Large Reported Max.  (Positive) Residual Density       1.94 eA-3
PLAT213_ALERT_2_C Atom C32             has ADP max/min Ratio .....        3.3 prolat
PLAT220_ALERT_2_C NonSolvent   Resd 1  C   Ueq(max)/Ueq(min) Range        5.5 Ratio
PLAT220_ALERT_2_C NonSolvent   Resd 1  O   Ueq(max)/Ueq(min) Range        3.3 Ratio
PLAT221_ALERT_2_C Solv./Anion  Resd 2  C   Ueq(max)/Ueq(min) Range        5.0 Ratio
PLAT222_ALERT_3_C NonSolvent Resd 1  H   Uiso(max)/Uiso(min) Range        4.9 Ratio
PLAT223_ALERT_4_C Solv./Anion  Resd 2  H   Ueq(max)/Ueq(min) Range        6.2 Ratio
PLAT223_ALERT_4_C Solv./Anion  Resd 3  H   Ueq(max)/Ueq(min) Range        4.2 Ratio
PLAT234_ALERT_4_C Large Hirshfeld Difference C30      --C62      .       0.18 Ang.
PLAT234_ALERT_4_C Large Hirshfeld Difference O63      --N4       .       0.17 Ang.
PLAT234_ALERT_4_C Large Hirshfeld Difference O66      --N4       .       0.16 Ang.
PLAT241_ALERT_2_C High   'MainMol' Ueq as Compared to Neighbors of        C48 Check
PLAT242_ALERT_2_C Low    'MainMol' Ueq as Compared to Neighbors of        C42 Check
PLAT242_ALERT_2_C Low    'MainMol' Ueq as Compared to Neighbors of        C61 Check
PLAT242_ALERT_2_C Low    'MainMol' Ueq as Compared to Neighbors of        C45 Check
PLAT242_ALERT_2_C Low    'MainMol' Ueq as Compared to Neighbors of        C47 Check
PLAT243_ALERT_4_C High   'Solvent' Ueq as Compared to Neighbors of         N4 Check
PLAT250_ALERT_2_C Large U3/U1 Ratio for Average U(i,j) Tensor ....        2.2 Note
PLAT329_ALERT_4_C Carbon Atom Hybridisation Unclear for ..........        C66 Check
PLAT329_ALERT_4_C Carbon Atom Hybridisation Unclear for ..........        C63 Check
PLAT341_ALERT_3_C Low Bond Precision on  C-C Bonds ...............    0.01455 Ang.
PLAT360_ALERT_2_C Short  C(sp3)-C(sp3) Bond  C32      - C42      .       1.39 Ang.
PLAT430_ALERT_2_C Short Inter D...A Contact  O22      ..O65      .       2.87 Ang.
                                                    x,y,z  =     1_555 Check
PLAT430_ALERT_2_C Short Inter D...A Contact  O53      ..O61      .       2.86 Ang.
                                                    x,y,z  =     1_555 Check
PLAT430_ALERT_2_C Short Inter D...A Contact  O57      ..O64      .       2.86 Ang.
                                                    x,y,z  =     1_555 Check
PLAT601_ALERT_2_C Unit Cell Contains Solvent Accessible VOIDS of .         31 Ang**3
PLAT906_ALERT_3_C Large K Value in the Analysis of Variance ......      2.032 Check
```

```
PLAT913_ALERT_3_C Missing # of Very Strong Reflections in FCF ....          4 Note
PLAT971_ALERT_2_C Check Calcd Resid. Dens.  1.38A   From V14             2.06 eA-3
PLAT971_ALERT_2_C Check Calcd Resid. Dens.  1.34A   From V18             2.03 eA-3
PLAT971_ALERT_2_C Check Calcd Resid. Dens.  1.03A   From O2              2.00 eA-3
PLAT971_ALERT_2_C Check Calcd Resid. Dens.  0.81A   From O65             1.89 eA-3
PLAT971_ALERT_2_C Check Calcd Resid. Dens.  1.20A   From C63             1.89 eA-3
PLAT971_ALERT_2_C Check Calcd Resid. Dens.  0.84A   From C67             1.75 eA-3
PLAT971_ALERT_2_C Check Calcd Resid. Dens.  1.32A   From O6              1.69 eA-3
PLAT971_ALERT_2_C Check Calcd Resid. Dens.  1.06A   From O54             1.61 eA-3
PLAT971_ALERT_2_C Check Calcd Resid. Dens.  1.34A   From V5              1.56 eA-3
PLAT977_ALERT_2_C Check Negative Difference Density on H42B             -0.49 eA-3
PLAT977_ALERT_2_C Check Negative Difference Density on H48B             -0.42 eA-3
PLAT977_ALERT_2_C Check Negative Difference Density on H52B             -0.46 eA-3
PLAT977_ALERT_2_C Check Negative Difference Density on H69C             -0.41 eA-3
PLAT977_ALERT_2_C Check Negative Difference Density on H71B             -0.35 eA-3
```

Alert level G

```
PLAT002_ALERT_2_G Number of Distance or Angle Restraints on AtSite          9 Note
PLAT003_ALERT_2_G Number of Uiso or Uij Restrained non-H Atoms ...         51 Report
PLAT012_ALERT_1_G No      _shelx_res_checksum Found in CIF ......     Please Check
PLAT042_ALERT_1_G Calc. and Reported MoietyFormula Strings  Differ     Please Check
PLAT083_ALERT_2_G SHELXL Second Parameter in WGHT  Unusually Large      95.93 Why ?
PLAT171_ALERT_4_G The CIF-Embedded .res File Contains EADP Records          1 Report
PLAT172_ALERT_4_G The CIF-Embedded .res File Contains DFIX Records          1 Report
PLAT176_ALERT_4_G The CIF-Embedded .res File Contains SADI Records          2 Report
PLAT178_ALERT_4_G The CIF-Embedded .res File Contains SIMU Records         12 Report
PLAT187_ALERT_4_G The CIF-Embedded .res File Contains RIGU Records          3 Report
PLAT300_ALERT_4_G Atom Site Occupancy of O61        Constrained at        0.5 Check
PLAT300_ALERT_4_G Atom Site Occupancy of O62        Constrained at        0.5 Check
PLAT300_ALERT_4_G Atom Site Occupancy of O63        Constrained at        0.5 Check
PLAT300_ALERT_4_G Atom Site Occupancy of O64        Constrained at        0.5 Check
PLAT300_ALERT_4_G Atom Site Occupancy of O65        Constrained at        0.5 Check
PLAT300_ALERT_4_G Atom Site Occupancy of O66        Constrained at        0.5 Check
PLAT302_ALERT_4_G Anion/Solvent/Minor-Residue Disorder (Resd  5  )        75% Note
PLAT343_ALERT_2_G Unusual sp?      Angle Range in Main Residue for        C66 Check
PLAT344_ALERT_2_G Unusual sp?       Angle Range in Solvent/Ion for        C63 Check
PLAT367_ALERT_2_G Long?  C(sp?)-C(sp?) Bond  C31      - C66      .       1.52 Ang.
PLAT432_ALERT_2_G Short Inter X...Y Contact  C31      ..C63              2.57 Ang.
                                                      x,y,z  =      1_555 Check
PLAT432_ALERT_2_G Short Inter X...Y Contact  C63      ..C66              1.76 Ang.
                                                      x,y,z  =      1_555 Check
PLAT432_ALERT_2_G Short Inter X...Y Contact  C66      ..C69              2.56 Ang.
                                                      x,y,z  =      1_555 Check
PLAT773_ALERT_2_G Check long C-C Bond in CIF: C63      --C66             1.76 Ang.
PLAT789_ALERT_4_G Atoms with Negative _atom_site_disorder_group  #          3 Check
PLAT794_ALERT_5_G Tentative Bond Valency for V16       (V)       .       5.17 Info
PLAT860_ALERT_3_G Number of Least-Squares Restraints .............        544 Note
PLAT978_ALERT_2_G Number C-C Bonds with Positive Residual Density.          0 Info
```

---

   1 **ALERT level A** = Most likely a serious problem - resolve or explain
   6 **ALERT level B** = A potentially serious problem, consider carefully
  46 **ALERT level C** = Check. Ensure it is not caused by an omission or oversight
  28 **ALERT level G** = General information/check it is not something unexpected

   3 ALERT type 1 CIF construction/syntax error, inconsistent or missing data
  48 ALERT type 2 Indicator that the structure model may be wrong or deficient
   8 ALERT type 3 Indicator that the structure quality may be low
  21 ALERT type 4 Improvement, methodology, query or suggestion
   1 ALERT type 5 Informative message, check

It is advisable to attempt to resolve as many as possible of the alerts in all categories. Often the minor alerts point to easily fixed oversights, errors and omissions in your CIF or refinement strategy, so attention to these fine details can be worthwhile. In order to resolve some of the more serious problems it may be necessary to carry out additional measurements or structure refinements. However, the purpose of your study may justify the reported deviations and the more serious of these should normally be commented upon in the discussion or experimental section of a paper or in the "special_details" fields of the CIF. checkCIF was carefully designed to identify outliers and unusual parameters, but every test has its limitations and alerts that are not important in a particular case may appear. Conversely, the absence of alerts does not guarantee there are no aspects of the results needing attention. It is up to the individual to critically assess their own results and, if necessary, seek expert advice.

Publication of your CIF in IUCr journals

A basic structural check has been run on your CIF. These basic checks will be run on all CIFs submitted for publication in IUCr journals (*Acta Crystallographica*, *Journal of Applied Crystallography*, *Journal of Synchrotron Radiation*); however, if you intend to submit to *Acta Crystallographica Section C* or *E* or *IUCrData*, you should make sure that full publication checks are run on the final version of your CIF prior to submission.

Publication of your CIF in other journals

Please refer to the *Notes for Authors* of the relevant journal for any special instructions relating to CIF submission.

PLATON version of 05/12/2020; check.def file version of 05/12/2020

Datablock sg20090_0m - ellipsoid plot

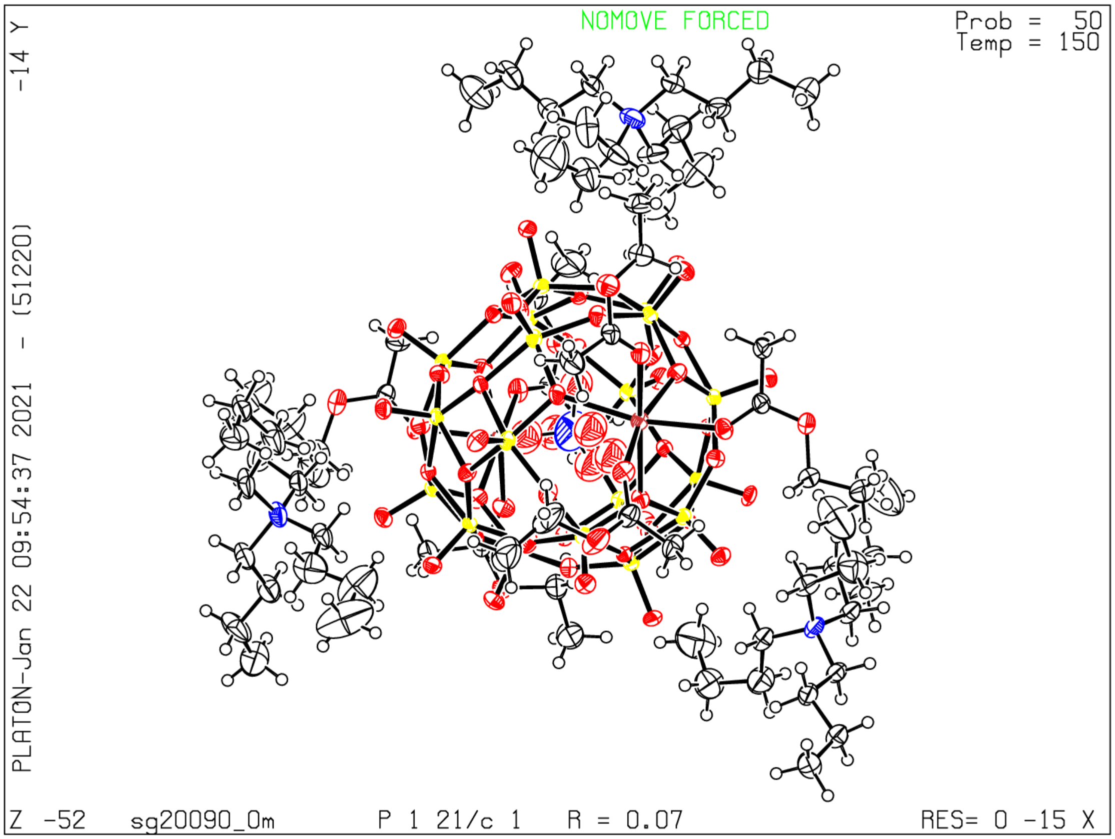

Supplement: Supplementary file 2 — Supporting Information [file ANIE-61-0-s006.pdf]
