# Supplementary material for: A General Access Route to High‐Nuclearity, Metal‐Functionalized Molecular Vanadium Oxides
Source: Angew Chem Int Ed Engl. 2022 Jan 17;61(9):e202114548. doi: 10.1002/anie.202114548 (PMC9302674; doi:10.1002/anie.202114548)

checkCIF/PLATON report

Structure factors have been supplied for datablock(s) ma18163_0m

THIS REPORT IS FOR GUIDANCE ONLY. IF USED AS PART OF A REVIEW PROCEDURE FOR PUBLICATION, IT SHOULD NOT REPLACE THE EXPERTISE OF AN EXPERIENCED CRYSTALLOGRAPHIC REFEREE.

No syntax errors found. CIF dictionary Interpreting this report

Datablock: ma18163_0m

Bond precision:	C-C = 0.0326 A		Wavelength=0.71073
Cell:	a=20.0983(5)	b=20.0983(5)	c=40.1754(8)
	alpha=90	beta=90	gamma=120
Temperature:	150 K		

	Calculated	Reported
Volume	14054.3(8)	14054.3(8)
Space group	P 32 2 1	P 32 2 1
Hall group	P 32 2"	P 32 2"
Moiety formula	C12 H18 Cu6 N8 O88 V30, 2(C16 H36 N), 2(C16 H21 N) [+ solvent]	C12 H18 Cu6 N6 O82 V30, 2(N O3), 2(C16 H36 N), 2(C16 H21 N)
Sum formula	C76 H132 Cu6 N12 O88 V30 [+ solvent]	C76 H132 Cu6 N12 O88 V30
Mr	4531.44	4531.37
Dx,g cm-3	1.606	1.606
Z	3	3
Mu (mm-1)	2.159	2.159
F000	6720.0	6720.0
F000’	6755.75	
h,k,lmax	25,25,50	25,25,50
Nref	19193[10438]	19192
Tmin,Tmax	0.732,0.871	0.679,0.745
Tmin’	0.701	

Correction method= # Reported T Limits: Tmin=0.679 Tmax=0.745
AbsCorr = MULTI-SCAN

Data completeness= 1.84/1.00 Theta(max)= 26.382

R(reflections)= 0.0404(15653) wR2(reflections)= 0.1111(19192)

S = 1.019 Npar= 981

The following ALERTS were generated. Each ALERT has the format
test-name_ALERT_alert-type_alert-level.
Click on the hyperlinks for more details of the test.

Alert level B

```
PLAT234_ALERT_4_B Large Hirshfeld Difference N6       --C29      .       0.30 Ang.
PLAT241_ALERT_2_B High   'MainMol' Ueq as Compared to Neighbors of        C22 Check
PLAT241_ALERT_2_B High   'MainMol' Ueq as Compared to Neighbors of        C34 Check
PLAT242_ALERT_2_B Low    'MainMol' Ueq as Compared to Neighbors of         N6 Check
PLAT260_ALERT_2_B Large Average Ueq of Residue Including        N6      0.311 Check
PLAT315_ALERT_2_B Singly Bonded Carbon Detected (H-atoms Missing).        C28 Check
PLAT315_ALERT_2_B Singly Bonded Carbon Detected (H-atoms Missing).        C37 Check
PLAT315_ALERT_2_B Singly Bonded Carbon Detected (H-atoms Missing).        C38 Check
PLAT341_ALERT_3_B Low Bond Precision on  C-C Bonds ..............     0.03256 Ang.
PLAT919_ALERT_3_B Reflection # Likely Affected by the Beamstop ...          1 Check
PLAT934_ALERT_3_B Number of (Iobs-Icalc)/Sigma(W) > 10 Outliers ..          2 Check
```

Alert level C

```
PLAT094_ALERT_2_C Ratio of Maximum / Minimum Residual Density ....       2.35 Report
PLAT213_ALERT_2_C Atom C19             has ADP max/min Ratio .....        3.1 prolat
PLAT213_ALERT_2_C Atom C31             has ADP max/min Ratio .....        3.7 prolat
PLAT215_ALERT_3_C Disordered V15       has ADP max/min Ratio .....        3.4 Note
PLAT223_ALERT_4_C Solv./Anion  Resd 2  H   Ueq(max)/Ueq(min) Range        4.4 Ratio
PLAT230_ALERT_2_C Hirshfeld Test Diff for    O39      --N1       .        6.2 s.u.
PLAT234_ALERT_4_C Large Hirshfeld Difference Cu3      --O23      .       0.16 Ang.
PLAT234_ALERT_4_C Large Hirshfeld Difference N2       --C32      .       0.16 Ang.
PLAT234_ALERT_4_C Large Hirshfeld Difference N6       --C22      .       0.22 Ang.
PLAT241_ALERT_2_C High   'MainMol' Ueq as Compared to Neighbors of        O67 Check
PLAT241_ALERT_2_C High   'MainMol' Ueq as Compared to Neighbors of        C32 Check
PLAT241_ALERT_2_C High   'MainMol' Ueq as Compared to Neighbors of        C29 Check
PLAT241_ALERT_2_C High   'MainMol' Ueq as Compared to Neighbors of        C30 Check
PLAT242_ALERT_2_C Low    'MainMol' Ueq as Compared to Neighbors of         N1 Check
PLAT242_ALERT_2_C Low    'MainMol' Ueq as Compared to Neighbors of         N2 Check
PLAT242_ALERT_2_C Low    'MainMol' Ueq as Compared to Neighbors of        C16 Check
PLAT242_ALERT_2_C Low    'MainMol' Ueq as Compared to Neighbors of        C14 Check
PLAT242_ALERT_2_C Low    'MainMol' Ueq as Compared to Neighbors of        C24 Check
PLAT242_ALERT_2_C Low    'MainMol' Ueq as Compared to Neighbors of        C27 Check
PLAT242_ALERT_2_C Low    'MainMol' Ueq as Compared to Neighbors of        C33 Check
PLAT242_ALERT_2_C Low    'MainMol' Ueq as Compared to Neighbors of        C36 Check
PLAT250_ALERT_2_C Large U3/U1 Ratio for Average U(i,j) Tensor ....        2.5 Note
PLAT260_ALERT_2_C Large Average Ueq of Residue Including        N5      0.105 Check
PLAT360_ALERT_2_C Short  C(sp3)-C(sp3) Bond  C9       - C11      .       1.42 Ang.
PLAT360_ALERT_2_C Short  C(sp3)-C(sp3) Bond  C20      - C23      .       1.43 Ang.
PLAT410_ALERT_2_C Short Intra H...H Contact  H20A     ..H29B     .       1.95 Ang.
                                                      x,y,z  =      1_555 Check
PLAT905_ALERT_3_C Negative K value in the Analysis of Variance ...     -0.019 Report
PLAT910_ALERT_3_C Missing # of FCF Reflection(s) Below Theta(Min).          8 Note
```

Alert level G

```
PLAT002_ALERT_2_G Number of Distance or Angle Restraints on AtSite         17 Note
PLAT003_ALERT_2_G Number of Uiso or Uij Restrained non-H Atoms ...         41 Report
PLAT012_ALERT_1_G No       _shelx_res_checksum Found in CIF ......     Please Check
PLAT014_ALERT_1_G No       _shelx_fab_checksum Found in CIF ......     Please Check
PLAT042_ALERT_1_G Calc. and Reported Moiety Formula Strings Differ     Please Check
PLAT083_ALERT_2_G SHELXL Second Parameter in WGHT  Unusually Large      13.53 Why ?
PLAT172_ALERT_4_G The CIF-Embedded .res File Contains DFIX Records          5 Report
PLAT176_ALERT_4_G The CIF-Embedded .res File Contains SADI Records          1 Report
PLAT177_ALERT_4_G The CIF-Embedded .res File Contains DELU Records         11 Report
PLAT178_ALERT_4_G The CIF-Embedded .res File Contains SIMU Records         11 Report
PLAT186_ALERT_4_G The CIF-Embedded .res File Contains ISOR Records          1 Report
PLAT187_ALERT_4_G The CIF-Embedded .res File Contains RIGU Records         12 Report
PLAT232_ALERT_2_G Hirshfeld Test Diff (M-X)  Cu3      --O67      .        7.8 s.u.
PLAT232_ALERT_2_G Hirshfeld Test Diff (M-X)  Cu4      --O10      .        5.8 s.u.
PLAT232_ALERT_2_G Hirshfeld Test Diff (M-X)  Cu4      --O30      .        5.4 s.u.
PLAT232_ALERT_2_G Hirshfeld Test Diff (M-X)  Cu4      --O67_a    .        7.3 s.u.
PLAT232_ALERT_2_G Hirshfeld Test Diff (M-X)  V15      --O51      .        6.4 s.u.
PLAT232_ALERT_2_G Hirshfeld Test Diff (M-X)  V15      --O67      .       12.8 s.u.
PLAT232_ALERT_2_G Hirshfeld Test Diff (M-X)  V16      --O10      .        5.4 s.u.
PLAT232_ALERT_2_G Hirshfeld Test Diff (M-X)  V16      --O47      .        5.8 s.u.
PLAT232_ALERT_2_G Hirshfeld Test Diff (M-X)  V16      --O67_a    .       10.8 s.u.
PLAT300_ALERT_4_G Atom Site Occupancy of Cu3        Constrained at        0.5 Check
PLAT300_ALERT_4_G Atom Site Occupancy of Cu4        Constrained at        0.5 Check
PLAT300_ALERT_4_G Atom Site Occupancy of V15        Constrained at        0.5 Check
PLAT300_ALERT_4_G Atom Site Occupancy of V16        Constrained at        0.5 Check
PLAT301_ALERT_3_G Main Residue  Disorder ..............(Resd  1  )         3% Note
PLAT343_ALERT_2_G Unusual sp?      Angle Range in Main Residue for        C32 Check
PLAT343_ALERT_2_G Unusual sp?      Angle Range in Main Residue for        C28 Check
PLAT343_ALERT_2_G Unusual sp3      Angle Range in Main Residue for        C29 Check
PLAT343_ALERT_2_G Unusual sp3      Angle Range in Main Residue for        C33 Check
PLAT343_ALERT_2_G Unusual sp?      Angle Range in Main Residue for        C34 Check
PLAT343_ALERT_2_G Unusual sp?      Angle Range in Main Residue for        C35 Check
PLAT343_ALERT_2_G Unusual sp?      Angle Range in Main Residue for        C36 Check
PLAT343_ALERT_2_G Unusual sp?      Angle Range in Main Residue for        C37 Check
PLAT343_ALERT_2_G Unusual sp?      Angle Range in Main Residue for        C38 Check
PLAT367_ALERT_2_G Long?  C(sp?)-C(sp?) Bond  C27      - C37      .       1.62 Ang.
PLAT367_ALERT_2_G Long?  C(sp?)-C(sp?) Bond  C28      - C33      .       1.52 Ang.
PLAT367_ALERT_2_G Long?  C(sp?)-C(sp?) Bond  C34      - C36      .       1.57 Ang.
PLAT367_ALERT_2_G Long?  C(sp?)-C(sp?) Bond  C35      - C36      .       1.57 Ang.
PLAT367_ALERT_2_G Long?  C(sp?)-C(sp?) Bond  C35      - C38      .       1.57 Ang.
PLAT606_ALERT_4_G Solvent Accessible VOID(S) in Structure ........          ! Info
PLAT779_ALERT_4_G Suspect or Irrelevant (Bond) Angle(s) in CIF ...       2.40 Deg.
              CU3  -O8   -V15     1_555   1_555   1_555 ......     #  171 Check
PLAT779_ALERT_4_G Suspect or Irrelevant (Bond) Angle(s) in CIF ...       0.90 Deg.
              CU4  -O10  -V16     1_555   1_555   1_555 ......     #  176 Check
PLAT779_ALERT_4_G Suspect or Irrelevant (Bond) Angle(s) in CIF ...       0.80 Deg.
              CU4  -O32  -V16     1_555   1_555   1_555 ......     #  203 Check
PLAT779_ALERT_4_G Suspect or Irrelevant (Bond) Angle(s) in CIF ...       2.50 Deg.
              CU3  -O38  -V15     1_555   1_555   1_555 ......     #  212 Check
PLAT779_ALERT_4_G Suspect or Irrelevant (Bond) Angle(s) in CIF ...       0.90 Deg.
              CU4  -O47  -V16     1_555   1_555   1_555 ......     #  220 Check
PLAT779_ALERT_4_G Suspect or Irrelevant (Bond) Angle(s) in CIF ...       2.70 Deg.
              CU3  -O51  -V15     1_555   1_555   1_555 ......     #  226 Check
PLAT779_ALERT_4_G Suspect or Irrelevant (Bond) Angle(s) in CIF ...       0.90 Deg.
              CU4  -O51  -V16     1_555   1_555   1_555 ......     #  229 Check
PLAT779_ALERT_4_G Suspect or Irrelevant (Bond) Angle(s) in CIF ...       0.60 Deg.
```

```
              V15  -O67  -CU3     1_555   1_555   1_555 ......     #  234 Check
PLAT789_ALERT_4_G Atoms with Negative _atom_site_disorder_group  #         2 Check
PLAT794_ALERT_5_G Tentative Bond Valency for Cu1       (II)      .      2.32 Info
PLAT794_ALERT_5_G Tentative Bond Valency for V1        (V)       .      5.03 Info
PLAT794_ALERT_5_G Tentative Bond Valency for V2        (V)       .      4.99 Info
PLAT794_ALERT_5_G Tentative Bond Valency for V6        (V)       .      5.16 Info
PLAT794_ALERT_5_G Tentative Bond Valency for V9        (V)       .      5.13 Info
PLAT794_ALERT_5_G Tentative Bond Valency for V10       (V)       .      5.06 Info
PLAT794_ALERT_5_G Tentative Bond Valency for V12       (V)       .      5.01 Info
PLAT794_ALERT_5_G Tentative Bond Valency for V13       (V)       .      5.00 Info
PLAT860_ALERT_3_G Number of Least-Squares Restraints .............       418 Note
PLAT913_ALERT_3_G Missing # of Very Strong Reflections in FCF ....         3 Note
PLAT978_ALERT_2_G Number C-C Bonds with Positive Residual Density.         0 Info
```

---

0 **ALERT level A** = Most likely a serious problem - resolve or explain
11 **ALERT level B** = A potentially serious problem, consider carefully
28 **ALERT level C** = Check. Ensure it is not caused by an omission or oversight
61 **ALERT level G** = General information/check it is not something unexpected

3 ALERT type 1 CIF construction/syntax error, inconsistent or missing data
55 ALERT type 2 Indicator that the structure model may be wrong or deficient
9 ALERT type 3 Indicator that the structure quality may be low
25 ALERT type 4 Improvement, methodology, query or suggestion
8 ALERT type 5 Informative message, check

It is advisable to attempt to resolve as many as possible of the alerts in all categories. Often the minor alerts point to easily fixed oversights, errors and omissions in your CIF or refinement strategy, so attention to these fine details can be worthwhile. In order to resolve some of the more serious problems it may be necessary to carry out additional measurements or structure refinements. However, the purpose of your study may justify the reported deviations and the more serious of these should normally be commented upon in the discussion or experimental section of a paper or in the "special_details" fields of the CIF. checkCIF was carefully designed to identify outliers and unusual parameters, but every test has its limitations and alerts that are not important in a particular case may appear. Conversely, the absence of alerts does not guarantee there are no aspects of the results needing attention. It is up to the individual to critically assess their own results and, if necessary, seek expert advice.

Publication of your CIF in IUCr journals

A basic structural check has been run on your CIF. These basic checks will be run on all CIFs submitted for publication in IUCr journals (*Acta Crystallographica*, *Journal of Applied Crystallography*, *Journal of Synchrotron Radiation*); however, if you intend to submit to *Acta Crystallographica Section C* or *E* or *IUCrData*, you should make sure that full publication checks are run on the final version of your CIF prior to submission.

Publication of your CIF in other journals

Please refer to the *Notes for Authors* of the relevant journal for any special instructions relating to CIF submission.

PLATON version of 13/07/2021; check.def file version of 13/07/2021

Datablock ma18163_0m - ellipsoid plot

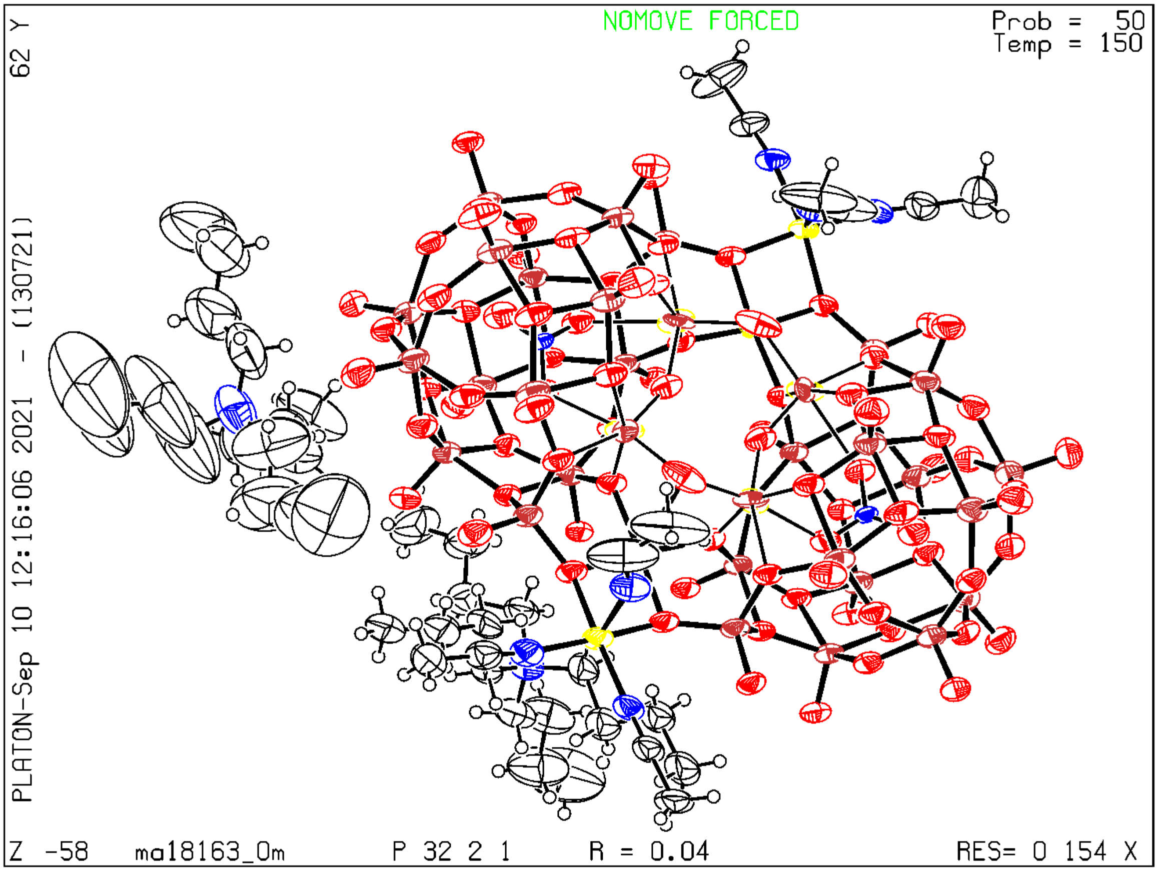

Supplement: Supplementary file 3 — Supporting Information [file ANIE-61-0-s003.pdf]
