# Supplementary material for: A General Access Route to High‐Nuclearity, Metal‐Functionalized Molecular Vanadium Oxides
Source: Angew Chem Int Ed Engl. 2022 Jan 17;61(9):e202114548. doi: 10.1002/anie.202114548 (PMC9302674; doi:10.1002/anie.202114548)

checkCIF/PLATON report

Structure factors have been supplied for datablock(s) sg20053_0m

THIS REPORT IS FOR GUIDANCE ONLY. IF USED AS PART OF A REVIEW PROCEDURE FOR PUBLICATION, IT SHOULD NOT REPLACE THE EXPERTISE OF AN EXPERIENCED CRYSTALLOGRAPHIC REFEREE.

No syntax errors found. CIF dictionary Interpreting this report

Datablock: sg20053_0m

```
Bond precision:   C-C = 0.0205 A          Wavelength=0.71073

Cell:              a=19.3285(14)      b=34.172(3)      c=21.0228(15)
                   alpha=90           beta=90          gamma=90
Temperature:       150 K
```

	Calculated	Reported
Volume	13885.4(19)	13885.3(17)
Space group	C 2 2 21	C 2 2 21
Hall group	C 2c 2	C 2c 2
Moiety formula	O46 V18, 4(C16 H36 N), C16 H30 N, N O3 [+ solvent]	O46 V18, 0.5(N2 O6), 4(C16 H36 N), C16 H30 N
Sum formula	C80 H174 N6 O49 V18 [+ solvent]	C80 H174 N6 O49 V18
Mr	2921.17	2921.16
Dx,g cm-3	1.397	1.397
Z	4	4
Mu (mm-1)	1.220	1.220
F000	6008.0	6008.0
F000’	6032.19	
h,k,lmax	24,42,26	24,42,26
Nref	14211[7647]	14173
Tmin,Tmax	0.706,0.813	0.587,0.745
Tmin’	0.692	

```
Correction method= # Reported T Limits: Tmin=0.587 Tmax=0.745
AbsCorr = MULTI-SCAN

Data completeness= 1.85/1.00        Theta(max)= 26.372
```

R(reflections)= 0.0594(9909)

wR2(reflections)= 0.1637(14173)

S = 1.013

Npar= 792

The following ALERTS were generated. Each ALERT has the format
test-name_ALERT_alert-type_alert-level.
Click on the hyperlinks for more details of the test.

Alert level A

```
PLAT216_ALERT_3_A Disordered O25       (An/Solv) ADP max/min Ratio       10.9 Note
```

Alert level B

```
PLAT341_ALERT_3_B Low Bond Precision on  C-C Bonds ...............     0.0205 Ang.
PLAT910_ALERT_3_B Missing # of FCF Reflection(s) Below Theta(Min).         27 Note
```

Alert level C

```
RINTA01_ALERT_3_C  The value of Rint is greater than 0.12
            Rint given   0.136
PLAT221_ALERT_2_C Solv./Anion  Resd 2  C   Ueq(max)/Ueq(min) Range        5.7 Ratio
PLAT221_ALERT_2_C Solv./Anion  Resd 3  C   Ueq(max)/Ueq(min) Range        4.2 Ratio
PLAT223_ALERT_4_C Solv./Anion  Resd 2  H   Ueq(max)/Ueq(min) Range        7.1 Ratio
PLAT223_ALERT_4_C Solv./Anion  Resd 3  H   Ueq(max)/Ueq(min) Range        5.2 Ratio
PLAT223_ALERT_4_C Solv./Anion  Resd 4  H   Ueq(max)/Ueq(min) Range        4.1 Ratio
PLAT234_ALERT_4_C Large Hirshfeld Difference O25      --N1        .      0.23 Ang.
PLAT242_ALERT_2_C Low    'MainMol' Ueq as Compared to Neighbors of       C29 Check
PLAT242_ALERT_2_C Low    'MainMol' Ueq as Compared to Neighbors of        C7 Check
PLAT242_ALERT_2_C Low    'MainMol' Ueq as Compared to Neighbors of        C8 Check
PLAT244_ALERT_4_C Low    'Solvent' Ueq as Compared to Neighbors of        N1 Check
PLAT250_ALERT_2_C Large U3/U1 Ratio for Average U(i,j) Tensor ....        2.2 Note
PLAT260_ALERT_2_C Large Average Ueq of Residue Including        N4      0.113 Check
PLAT360_ALERT_2_C Short  C(sp3)-C(sp3) Bond  C29      - C32       .      1.39 Ang.
PLAT360_ALERT_2_C Short  C(sp3)-C(sp3) Bond  C7       - C18       .      1.42 Ang.
PLAT790_ALERT_4_C Centre of Gravity not Within Unit Cell: Resd.  #          1 Note
              O46 V18
PLAT934_ALERT_3_C Number of (Iobs-Icalc)/Sigma(W) > 10 Outliers ..          1 Check
PLAT975_ALERT_2_C Check Calcd Resid. Dens.  1.07A   From C36             0.91 eA-3
```

Alert level G

```
PLAT002_ALERT_2_G Number of Distance or Angle Restraints on AtSite         15 Note
PLAT003_ALERT_2_G Number of Uiso or Uij Restrained non-H Atoms ...         51 Report
PLAT012_ALERT_1_G No       _shelx_res_checksum Found in CIF ......     Please Check
PLAT014_ALERT_1_G No       _shelx_fab_checksum Found in CIF ......     Please Check
PLAT020_ALERT_3_G The Value of Rint is Greater Than 0.12 .........      0.136 Report
PLAT042_ALERT_1_G Calc. and Reported Moiety Formula Strings Differ     Please Check
PLAT152_ALERT_1_G The Supplied and Calc. Volume s.u. Differ by ...          2 Units
PLAT172_ALERT_4_G The CIF-Embedded .res File Contains DFIX Records          7 Report
PLAT177_ALERT_4_G The CIF-Embedded .res File Contains DELU Records         17 Report
PLAT178_ALERT_4_G The CIF-Embedded .res File Contains SIMU Records         17 Report
PLAT186_ALERT_4_G The CIF-Embedded .res File Contains ISOR Records          1 Report
PLAT300_ALERT_4_G Atom Site Occupancy of N4         Constrained at        0.5 Check
```

```
PLAT300_ALERT_4_G Atom Site Occupancy of C33        Constrained at        0.5 Check
PLAT300_ALERT_4_G Atom Site Occupancy of C34        Constrained at        0.5 Check
PLAT300_ALERT_4_G Atom Site Occupancy of C35        Constrained at        0.5 Check
PLAT300_ALERT_4_G Atom Site Occupancy of C36        Constrained at        0.5 Check
PLAT300_ALERT_4_G Atom Site Occupancy of C37        Constrained at        0.5 Check
PLAT300_ALERT_4_G Atom Site Occupancy of C38        Constrained at        0.5 Check
PLAT300_ALERT_4_G Atom Site Occupancy of C39        Constrained at        0.5 Check
PLAT300_ALERT_4_G Atom Site Occupancy of C40        Constrained at        0.5 Check
PLAT300_ALERT_4_G Atom Site Occupancy of C41        Constrained at        0.5 Check
PLAT300_ALERT_4_G Atom Site Occupancy of C42        Constrained at        0.5 Check
PLAT300_ALERT_4_G Atom Site Occupancy of C43        Constrained at        0.5 Check
PLAT300_ALERT_4_G Atom Site Occupancy of C44        Constrained at        0.5 Check
PLAT300_ALERT_4_G Atom Site Occupancy of C45        Constrained at        0.5 Check
PLAT300_ALERT_4_G Atom Site Occupancy of C46        Constrained at        0.5 Check
PLAT300_ALERT_4_G Atom Site Occupancy of C47        Constrained at        0.5 Check
PLAT300_ALERT_4_G Atom Site Occupancy of C48        Constrained at        0.5 Check
PLAT300_ALERT_4_G Atom Site Occupancy of H33A       Constrained at        0.5 Check
PLAT300_ALERT_4_G Atom Site Occupancy of H33B       Constrained at        0.5 Check
PLAT300_ALERT_4_G Atom Site Occupancy of H34A       Constrained at        0.5 Check
PLAT300_ALERT_4_G Atom Site Occupancy of H34B       Constrained at        0.5 Check
PLAT300_ALERT_4_G Atom Site Occupancy of H35A       Constrained at        0.5 Check
PLAT300_ALERT_4_G Atom Site Occupancy of H35B       Constrained at        0.5 Check
PLAT300_ALERT_4_G Atom Site Occupancy of H37A       Constrained at        0.5 Check
PLAT300_ALERT_4_G Atom Site Occupancy of H37B       Constrained at        0.5 Check
PLAT300_ALERT_4_G Atom Site Occupancy of H38A       Constrained at        0.5 Check
PLAT300_ALERT_4_G Atom Site Occupancy of H38B       Constrained at        0.5 Check
PLAT300_ALERT_4_G Atom Site Occupancy of H39A       Constrained at        0.5 Check
PLAT300_ALERT_4_G Atom Site Occupancy of H39B       Constrained at        0.5 Check
PLAT300_ALERT_4_G Atom Site Occupancy of H40A       Constrained at        0.5 Check
PLAT300_ALERT_4_G Atom Site Occupancy of H40B       Constrained at        0.5 Check
PLAT300_ALERT_4_G Atom Site Occupancy of H40C       Constrained at        0.5 Check
PLAT300_ALERT_4_G Atom Site Occupancy of H41A       Constrained at        0.5 Check
PLAT300_ALERT_4_G Atom Site Occupancy of H41B       Constrained at        0.5 Check
PLAT300_ALERT_4_G Atom Site Occupancy of H42A       Constrained at        0.5 Check
PLAT300_ALERT_4_G Atom Site Occupancy of H42B       Constrained at        0.5 Check
PLAT300_ALERT_4_G Atom Site Occupancy of H43A       Constrained at        0.5 Check
PLAT300_ALERT_4_G Atom Site Occupancy of H43B       Constrained at        0.5 Check
PLAT300_ALERT_4_G Atom Site Occupancy of H44A       Constrained at        0.5 Check
PLAT300_ALERT_4_G Atom Site Occupancy of H44B       Constrained at        0.5 Check
PLAT300_ALERT_4_G Atom Site Occupancy of H46A       Constrained at        0.5 Check
PLAT300_ALERT_4_G Atom Site Occupancy of H46B       Constrained at        0.5 Check
PLAT300_ALERT_4_G Atom Site Occupancy of H47A       Constrained at        0.5 Check
PLAT300_ALERT_4_G Atom Site Occupancy of H47B       Constrained at        0.5 Check
PLAT300_ALERT_4_G Atom Site Occupancy of H48A       Constrained at        0.5 Check
PLAT300_ALERT_4_G Atom Site Occupancy of H48B       Constrained at        0.5 Check
PLAT300_ALERT_4_G Atom Site Occupancy of H48C       Constrained at        0.5 Check
PLAT300_ALERT_4_G Atom Site Occupancy of O25        Constrained at        0.5 Check
PLAT300_ALERT_4_G Atom Site Occupancy of O26        Constrained at        0.5 Check
PLAT300_ALERT_4_G Atom Site Occupancy of O27        Constrained at        0.5 Check
PLAT302_ALERT_4_G Anion/Solvent/Minor-Residue Disorder (Resd  4  )       100% Note
PLAT302_ALERT_4_G Anion/Solvent/Minor-Residue Disorder (Resd  5  )        75% Note
PLAT304_ALERT_4_G Non-Integer Number of Atoms in ..... (Resd  4  )      23.50 Check
PLAT315_ALERT_2_G Singly Bonded Carbon Detected (H-atoms Missing).        C36 Check
PLAT315_ALERT_2_G Singly Bonded Carbon Detected (H-atoms Missing).        C45 Check
PLAT395_ALERT_2_G Deviating  X-O-Y    Angle From 120 for O26           53.9 Degree
PLAT395_ALERT_2_G Deviating  X-O-Y    Angle From 120 for O27           56.2 Degree
PLAT413_ALERT_2_G Short Inter XH3 .. XHn     H32A     ..H37B     .       2.12 Ang.
```

```
                                           -2-x,y,-3/2-z  =      4_353 Check
PLAT413_ALERT_2_G Short Inter XH3 .. XHn     H32B     ..H38B     .       1.91 Ang.
                                           -2-x,y,-3/2-z  =      4_353 Check
PLAT413_ALERT_2_G Short Inter XH3 .. XHn     H31C     ..H48B     .       2.11 Ang.
                                               x,-y,-2-z  =      3_553 Check
PLAT432_ALERT_2_G Short Inter X...Y Contact  O7       ..C47              3.01 Ang.
                                                   x,y,z  =      1_555 Check
PLAT432_ALERT_2_G Short Inter X...Y Contact  C18      ..C45              2.93 Ang.
                                               x,-y,-1-z  =      3_554 Check
PLAT432_ALERT_2_G Short Inter X...Y Contact  C35      ..C45              2.15 Ang.
                                           -2-x,y,-3/2-z  =      4_353 Check
PLAT432_ALERT_2_G Short Inter X...Y Contact  C39      ..C45              2.50 Ang.
                                           -2-x,y,-3/2-z  =      4_353 Check
PLAT432_ALERT_2_G Short Inter X...Y Contact  C41      ..C45              2.16 Ang.
                                           -2-x,y,-3/2-z  =      4_353 Check
PLAT605_ALERT_4_G Largest Solvent Accessible VOID in the Structure        199 A**3
PLAT790_ALERT_4_G Centre of Gravity not Within Unit Cell: Resd.  #          2 Note
              C16 H36 N
PLAT790_ALERT_4_G Centre of Gravity not Within Unit Cell: Resd.  #          3 Note
              C16 H36 N
PLAT790_ALERT_4_G Centre of Gravity not Within Unit Cell: Resd.  #          4 Note
              C16 H30 N
PLAT790_ALERT_4_G Centre of Gravity not Within Unit Cell: Resd.  #          5 Note
              N O3
PLAT850_ALERT_4_G Check Flack Parameter Exact Value 0.00 with s.u.       0.02 Check
PLAT860_ALERT_3_G Number of Least-Squares Restraints ............         304 Note
PLAT913_ALERT_3_G Missing # of Very Strong Reflections in FCF ....          2 Note
PLAT978_ALERT_2_G Number C-C Bonds with Positive Residual Density.          0 Info
```

```
   1 ALERT level A = Most likely a serious problem - resolve or explain
   2 ALERT level B = A potentially serious problem, consider carefully
  18 ALERT level C = Check. Ensure it is not caused by an omission or oversight
  85 ALERT level G = General information/check it is not something unexpected

   4 ALERT type 1 CIF construction/syntax error, inconsistent or missing data
  25 ALERT type 2 Indicator that the structure model may be wrong or deficient
   8 ALERT type 3 Indicator that the structure quality may be low
  69 ALERT type 4 Improvement, methodology, query or suggestion
   0 ALERT type 5 Informative message, check
```

It is advisable to attempt to resolve as many as possible of the alerts in all categories. Often the minor alerts point to easily fixed oversights, errors and omissions in your CIF or refinement strategy, so attention to these fine details can be worthwhile. In order to resolve some of the more serious problems it may be necessary to carry out additional measurements or structure refinements. However, the purpose of your study may justify the reported deviations and the more serious of these should normally be commented upon in the discussion or experimental section of a paper or in the "special_details" fields of the CIF. checkCIF was carefully designed to identify outliers and unusual parameters, but every test has its limitations and alerts that are not important in a particular case may appear. Conversely, the absence of alerts does not guarantee there are no aspects of the results needing attention. It is up to the individual to critically assess their own results and, if necessary, seek expert advice.

Publication of your CIF in IUCr journals

A basic structural check has been run on your CIF. These basic checks will be run on all CIFs submitted for publication in IUCr journals (*Acta Crystallographica*, *Journal of Applied Crystallography*, *Journal of Synchrotron Radiation*); however, if you intend to submit to *Acta Crystallographica Section C* or *E* or *IUCrData*, you should make sure that full publication checks are run on the final version of your CIF prior to submission.

Publication of your CIF in other journals

Please refer to the *Notes for Authors* of the relevant journal for any special instructions relating to CIF submission.

Validation response form

Please find below a validation response form (VRF) that can be filled in and pasted into your CIF.

```
# start Validation Reply Form
_vrf_PLAT216_sg20053_0m
;
PROBLEM: Disordered O25       (An/Solv) ADP max/min Ratio       10.9 Note
RESPONSE: ...
;
_vrf_PLAT341_sg20053_0m
;
PROBLEM: Low Bond Precision on  C-C Bonds ...............     0.0205 Ang.
RESPONSE: ...
;
_vrf_PLAT910_sg20053_0m
;
PROBLEM: Missing # of FCF Reflection(s) Below Theta(Min).         27 Note
RESPONSE: ...
;
# end Validation Reply Form
```

PLATON version of 13/07/2021; check.def file version of 13/07/2021

Datablock sg20053_0m - ellipsoid plot

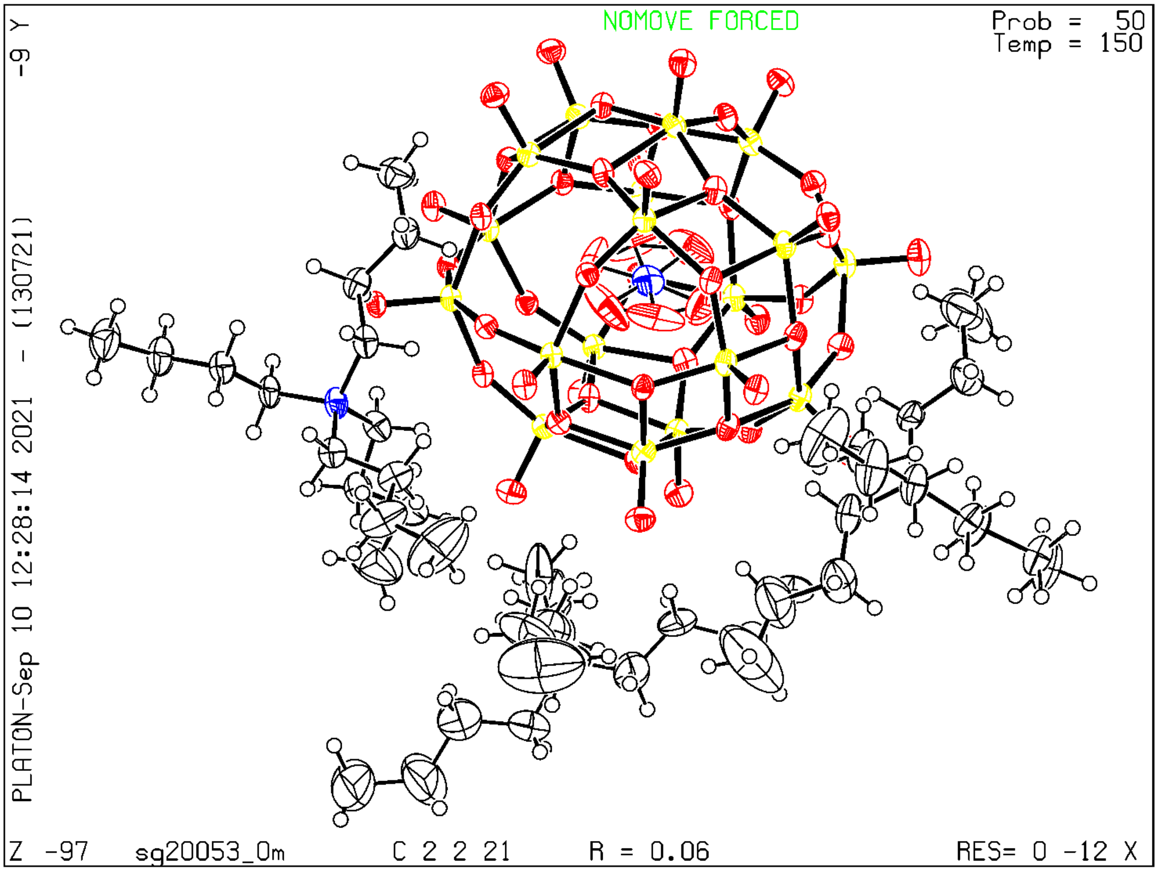

Supplement: Supplementary file 4 — Supporting Information [file ANIE-61-0-s005.pdf]
