# Supplementary material for: A General Access Route to High‐Nuclearity, Metal‐Functionalized Molecular Vanadium Oxides
Source: Angew Chem Int Ed Engl. 2022 Jan 17;61(9):e202114548. doi: 10.1002/anie.202114548 (PMC9302674; doi:10.1002/anie.202114548)
Supplement: Supplementary file 6 — Supporting Information [file ANIE-61-0-s001.pdf]

Angewandte Chemie
Eine Zeitschrift der Gesellschaft Deutscher Chemiker

Supporting Information

A General Access Route to High-Nuclearity, Metal-Functionalized Molecular Vanadium Oxides

S. Greiner, J. Hettig, A. Laws, K. Baumgärtner, J. Bustos, A.-C. Pöppler, A. H. Clark, M. Nyman, C. Streb*, M. Anjass**

1. Instrumentation

Single-crystal X-ray diffraction (scXRD) was measured on a Bruker APEX-II CCD Single-crystal X-ray diffractometer equipped with a graphite monochromator using $Mo_{K\alpha}$ radiation (wavelength $\lambda(Mo_{K\alpha})$ = 0.71073 Å).

Attenuated total reflectance-Fourier-transformed infrared spectroscopy (ATR-FT-IR) was recorded using a PerkinElmer Spectrum Two spectrometer in a range between 4000 and 500 cm^{-1}.

UV-vis spectroscopy was performed on a Varian Cary 50 spectrophotometer in a standard cuvette (d = 10.0 mm)

Elemental Analysis: CHNS analysis was performed on a Vario MICRO cube. Inductively coupled plasma optical emission spectroscopy (ICP-OES) was performed on a Spectro Arcos FHS12.

^{1}H NMR spectroscopy was performed on a Bruker Avance III 400 MHz instrument at a sample temperature of 23 °C.

Small-angle X-ray diffraction (SAXS) SAXS data were collected on an Anton Parr SAXSess instrument utilizing Cu-Ka radiation and line collimation. Data were recorded on an image plate in the range of 0.08-2.5 $Å^{-1}$. Sample to image plate distance of 26.1 cm. Solutions were measured in 1.5 mm glass capillaries. Neat acetonitrile was used as the background, and scattering was measured for 30 minutes. SAXSQUANT software was used for data collection and processing (normalization, primary beam removal, and background subtraction). Simulated scattering curve of the V_{10}-dimer were generated using SolX utilizing structural files (.xyz) containing the selected portion of the structure with no symmetry operations.[1]

Magic angle spinning-^{51}V NMR spectroscopy All vanadium solid-state NMR spectra were recorded at room temperature through direct excitation of the ^{51}V nuclei using a 4 mm Bruker double resonance iProbe at a 9.4 T magnet. ^{51}V 90° pulse lengths were calibrated using V_2O_5. To capture the wide sideband pattern, variable offset stepwise acquisition was used. The spectral widths of each individual spectrum varied between 1200 and 2400 ppm; the offset was changed in steps of 700-1800 ppm. To identify isotropic chemical shifts all samples were measured at two or more different MAS frequencies.

Thermogravimetric analysis (TGA) was carried out on a NETZSCH TG 209F1 analyzer at a heating rate of 10.0 K min^{-1} in a range between 30 and 600 °C under O_2/N_2 in an Al crucible.

Mass spectrometry: Electrospray-ionization mass spectrometry (ESI-MS) was performed using *ultra-high-resolution* time-of-flight (UHR-TOF) Bruker Daltonik maXis mass spectrometer in negative ion detection mode. Measurement conditions: source voltage: 4 kV, sample flow rate: 500 µl/h, drying gas temperature (N2): 180 °C.

X-ray photoelectron spectroscopy (XPS) was performed on Physical Electronics PHI 5800 spectrometer using monochromatized $Al_{K\alpha}$ (1486.6 eV) radiation. The measurements were performed with a detection angle of 45 °C, using pass energies at the analyzer of 93.9 and 29.35 eV for survey and detail spectra, respectively. The samples were neutralized with electrons from a flood gun (current 3 µA) to compensate for charging effects at the surface.

X-ray absorption spectroscopy was carried out as ex-situ experiment at the SuperXAS beamline of the Swiss Light Source. The storage ring operated at 2.4 GeV in top-up mode with a ring current of 400 mA. The polychromatic X-ray beam resulting from a 2.9 Tesla bending magnet was collimated by a Si-coated mirror at 2.5 mrad (which also served to reduce higher-order harmonics) and subsequently monochromatized by a Si(111) channel-cut monochromator. Data were collected in transmission geometry using the quick-scanning extended X-ray absorption fine-structure spectroscopy (QEXAFS) mode at 1 Hz monochromator oscillation frequency. Ionization chambers 15 cm long were filled with 1 bar N_2. A V reference foil mounted between the second and third ionization chambers was measured simultaneously for absolute energy calibration. The samples were prepared as 13 mm pellets with the samples diluted with cellulose to obtain an edge jump close to 1. The data were processed using ProQEXAFS.[2] Fitting of the EXAFS data was performed by the program Artemis[3], which is based on the FEFF[4] code.

2. Electrochemical characterization

DC cyclic voltammetry (CV) experiments were performed on a Pine Research WaveDriver electrochemical workstation equipped with a standard three-electrode arrangement: working electrode: glassy carbon electrode (d = 3.0 mm), quasi reference electrode: Ag wire (in a glass frit containing electrolyte solution), counter electrode: Pt wire. All potentials are quoted relative to the ferrocene/ferrocenium internal standard.
All experiments were performed in dry MeCN using $n\text{Bu}_4\text{NPF}_6$ (0.1 M) as supporting electrolyte. The solutions were purged with argon for at least 15 min to remove O_2 and kept under a slight positive Ar pressure while performing the experiments.

3. Experimental procedures

All chemicals were purchased from Sigma Aldrich, VWR or Alfa Aesar and were of reagent grade. The chemicals were used without further purification unless stated otherwise. $(n\text{Bu}_4\text{N})_3[\text{H}_3\text{V}_{10}\text{O}_{28}]$ was prepared according to literature.[5]

Synthesis of $(n\text{Bu}_4\text{N})[\text{V}_{10}\text{O}_{24}]$ (**1**):

$(n\text{Bu}_4\text{N})_3[\text{H}_3\text{V}_{10}\text{O}_{28}]$[5] (800 mg) was heated at 195 °C for 2 hours under air after heating the sample at a rate of 200 K h^{-1}. After slow cooling of the sample in the oven, the orange powder had turned dark brown and remained totally soluble in acetonitrile.

Characteristic IR bands (in cm^{-1}): 2960 (m), 2932 (m), 2871 (m), 1628 (w), 1583 (w), 1480 (m), 1462 (m), 1379 (m), 1151 (w), 1107 (w), 1063 (w), 997 (vs), 922 (m), 888 (s), 840 (s), 694 (s), 651 (s), 592 (vs).

ICP-OES in wt.-% (calcd.): V 36.71 (36.58).

Elemental analysis in wt.-% (calcd.): C 27.50 (27.88), H 4.90 (5.26), N 2.05 (2.03).

^{1}H NMR (400 MHz, MeCN-d_3): δ (ppm): 3.15 (broad, 16H, $(n\text{Bu}_4\text{N})^+$), 1.62 (broad, 16H, $(n\text{Bu}_4\text{N})^+$), 1.40 (broad, 16H, $(n\text{Bu}_4\text{N})^+$), 0.99 (broad, 24H, $(n\text{Bu}_4\text{N})^+$).

Synthesis of $(n\text{Bu}_4\text{N})_5[\text{V}_{18}\text{O}_{46}(\text{NO}_3)]$ (**{V$_{18}$}**):

1 (200 mg, 0.145 mmol, 1 eq.) was dissolved in acetonitrile (20 ml) and $(n\text{Bu}_4\text{N})(\text{NO}_3)$ (801.4 mg, 2.63 mmol, 18 eq.) were added. The resulting brown-red solution was stirred overnight. The formed precipitate was filtered off and addition of ethyl acetate (60ml) yielded dark brown crystals suitable for X-ray diffraction after 1 month.

Yield: 176 mg (55 µmol, 69% based on V).

Characteristic IR bands (in cm^{-1}): 2958 (m), 2933 (m), 2870 (m), 1626 (w), 1481 (m), 1463 (m), 1378 (w), 1360 (m), 1340 (m), 1152 (w), 1108 (w), 1066 (w), 988 (vs), 878 (w), 826 (s), 775 (s), 622 (vs), 567 (s), 523 (m).

UV/vis (in MeCN): ε_{225} = 87370 M^{-1} cm^{-1}, ε_{887} = 3857 M^{-1} cm^{-1}.

ICP-OES in wt.-% (calcd.): V 31.06 (30.89).

Synthesis of $(n\text{Bu}_4\text{N})_4[\text{Cu}_6\text{V}_{30}\text{O}_{82}(\text{NO}_3)_2(\text{CH}_3\text{CN})_6]$ (**{Cu$_6$V$_{30}$}**):

1 (400 mg, 0.29 mmol, 1eq.) and $Cu(NO_3)_2 \cdot 3\,H_2O$ (1272 mg, 5.3 mmol, 18 eq.) were dissolved in acetonitrile (40 ml) and stirred overnight and the yellow precipitate was filtered off. Addition of ethyl acetate (130 ml) yielded dark brown needles suitable for X-ray diffraction after ca. one week.

Yield: 190 mg (42 µmol, 43% based on V)

Characteristic IR bands (in cm^{-1}): 3334 (b), 2963 (m), 2935 (m), 2874 (m), 1730 (w), 1695 (w), 1650 (w), 1482 (w), 1463 (w), 1376 (s), 1363 (s), 1245 (w), 990 (vs), 935 (m), 914 (m), 831 (vs), 710 (vs), 571 (vs).

ICP-OES in wt.-% (calcd.): Cu 8.08 (8.36), V 32.23 (33.50).

Synthesis of $(n\text{Bu}_4\text{N})_3[\text{Ca}_2(\text{C}_4\text{H}_8\text{O}_2)_6\text{V}_{18}\text{O}_{48}(\text{NO}_3)]$ (**{Ca$_2$V$_{18}$}**):

$Ca(NO_3)_2 \cdot 4\,H_2O$ (620 mg, 2.62 mmol, 18 eq.) was dissolved in a few drops of water and added to a solution of **1** (200 mg, 0.145 mmol, 1 eq.) in acetonitrile (40 ml). The resulting greenish-brown solution was vigorously stirred overnight. After removal of the formed precipitate, diffusion of ethyl acetate into the clear reaction mixture gave orange crystals after 2 weeks.

Yield: 66 mg (21.4 µmol, 27% based on V).

Characteristic IR bands (in cm^{-1}): 3492 (b), 2962 (m), 2935 (m), 2871 (m), 1710 (m), 1691 (s), 1619 (w), 1480 (m), 1459 (m), 1428 (w), 1375 (m), 1358 (w), 1339 (m), 1287 (s), 1267 (m), 1155 (w), 1110 (w), 1043 (m), 991 (vs), 851 (vs), 802 (s), 740 (vs), 627 (vs), 600 (vs).

UV/vis (in MeCN): ε_{237} = 94938 M^{-1} cm^{-1}; ε_{378} = 21796 M^{-1} cm^{-1}; ε_{832} = 356 M^{-1} cm^{-1}.

ICP-OES in wt.-% (calcd.): Ca 2.90 (2.57), V 30.28 (29.35).

Elemental analysis in wt.-% (calcd.): C 27.16 (28.45), H 4.74 (5.13), N 2.18 (2.24).

4. Crystallographic section

Suitable single-crystals were mounted onto a microloop using Fomblin oil. X-ray diffraction intensity data were measured at 150 K on a Bruker D8 QUEST diffractometer λ($Mo_{Kα}$ = 0.71073 Å) equipped with a graphite monochromator. Structure solution was carried out using SHELX-2013[6] package through OLEX2[7] Corrections for incident and diffracted beam absorption effects were applied using empirical methods.[8] Structures were solved by a combination of direct methods and difference Fourier syntheses and refined against F^2 by the full matrix least-squares technique. Non-hydrogen atoms were refined anisotropically. Hydrogen atoms were added to carbon atoms using a riding model. The geometry and anisotropic refinement of the ethyl acetate molecules and tetrabutylammonium cations were restrained using SIMU and RIGU. The nitrate template was restrained using SADI and EADP. The metal oxo framework was refined fully anisotropically and no restraints were used. CIF files of novel compounds can be obtained free of charge from the CCDC.

Table S1 Crystallographic parameters for **{Ca_2V_{18}}**, **{V_{18}}**, and **{Cu_6V_{30}}**.

Compound code	**{Ca_2V_{18}}**	**{V_{18}}**	**{Cu_6V_{30}}**
CCDC code	2058109	2109076	2109067
Empirical formula	$C_{72}H_{156}Ca_2N_4O_{63}V_{18}$	$C_{80}H_{174}N_6O_{49}V_{18}$	$C_{76}H_{132}Cu_6N_{12}O_{88}V_{30}$
Formula weight / g mol^{-1}	3083.08	2921.16	4531.37
Temperature / K	150	150	150
Wavelength / nm	0.71073	0.71073	0.71073
Crystal system	Monoclinic	Orthorhombic	Trigonal
Space group	$P2_1/c$	$C222_1$	$P3_221$
Unit cell dimensions / Å	a = 27.641(2)<br>b = 16.3250(14)<br>c = 28.068(2)	a = 19.3285(14)<br>b = 34.172(3)<br>c = 21.0228(15)	a = 20.0983(5)<br>b = 20.0983(5)<br>c = 40.1754(8)
Unit cell angles / °	α = β = 90<br>γ = 105.915(2)	α = β = γ = 90	α = β = 90<br>γ = 120
Volume / Å^3	12179.9(17)	13855.3(17)	14054.3(8)
Z	4	4	3
Density (calcd.) / g cm^{-3}	1.681	1.397	1.606
Absorption coefficient μ / mm^{-1}	1.485	1.220	2.159
F(000)	6296.0	6008.0	6720.0
2Θ range for data collection / °	6.872 to 53.466	7.146 to 52.744	4.054 to 52.764
Index ranges	-34 ≤ h ≤ 34, -20 ≤ k ≤ 20, -35 ≤ l ≤ 33	-24 ≤ h ≤ 23, -42 ≤ k ≤ 42, -23 ≤ l ≤ 26	-25 ≤ h ≤ 25, -25 ≤ k ≤ 22, -50 ≤ l ≤ 50
No. reflections	309830	54905	127987
Independent reflections	25799 [R_{int} = 0.1285, R_{sigma} = 0.0526]	14173 [R_{int} = 0.1362, R_{sigma} = 0.1132]	19192 [R_{int} = 0.595, R_{sigma} = 0.0362]
Data / restraints / parameters	25799/544/1453	14173/304/792	19192/418/981
Goodness-of-fit	1.112	1.013	1.019
Final R indices [I>2sigma(I)]	R_1 = 0.0740, wR_2 = 0.1835	R_1 = 0.0594, wR_2 = 0.1414	R_1 = 0.0404, wR_2 = 0.1016
R indices (all data)	R_1 = 0.1023, wR_2 = 0.2073	R_1 = 0.1006, wR_2 = 0.1637	R_1 = 0.0551, wR_2 = 0.1111
Largest diff. peak and hole	1.94/-0.83	0.82/-0.42	0.84/-0.36
Flack parameter	-	0.00(2)	0.004(6)

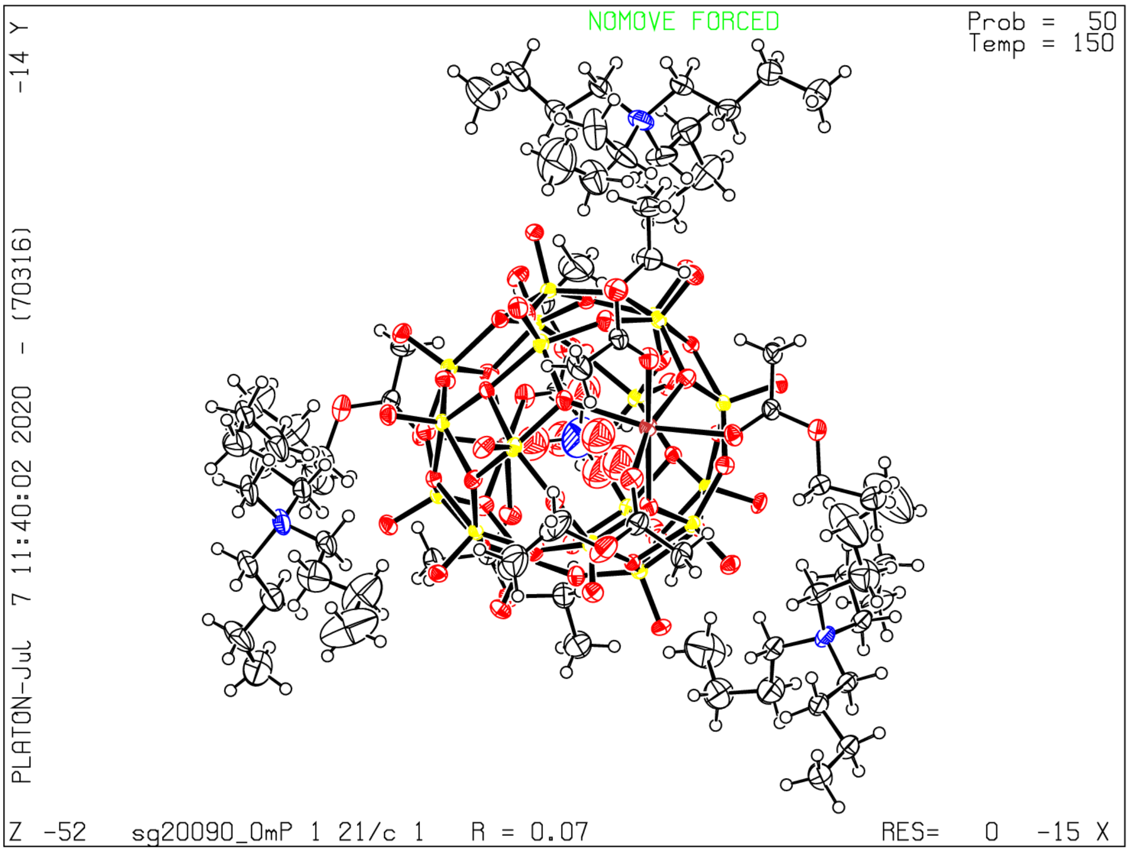

Figure S1 ORTEP-representation of **{Ca_2V_{18}}**, probability ellipsoids shown at 50%.

Table S2 Unit cell data comparison of the known compounds **{V_{18}}** and **{Cu_6V_{30}}** with literature data.

	**{Cu_6V_{30}} observed**	**{Cu_6V_{30}} reported**[9] **(CCDC = 890307)**	**(V_{18}} observed**	**(V_{18}} reported**[10]
**a / Å**	20.0983(5)	20.411(3)	19.3285(14)	19.7196(16)
**b / Å**	20.0983(5)	20.411(3)	21.0228(15)	21.0455(17)
**c / Å**	40.1754(8)	40.340(5)	34.172(3)	33.937(3)
**α / °**	90	90	90	90
**β / °**	90	90	90	90
**γ / °**	120	120	90	90

5. Infrared Spectroscopy

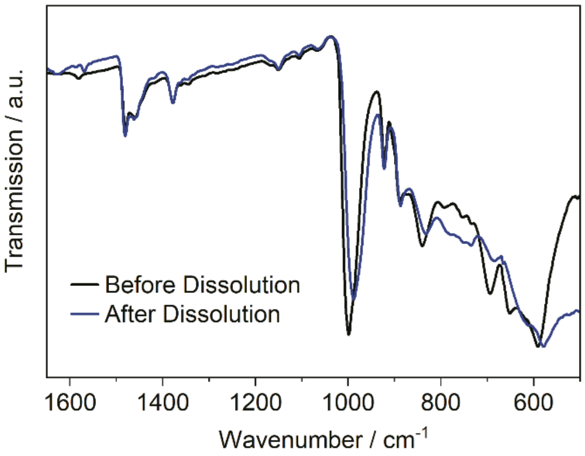

Figure S2 Infrared spectrum of **1** before (black) and after (blue) dissolving in acetonitrile and evaporating the solvent. The changes in the V-O-fingerprint region (1000 – 500 cm^{-1}) indicate immediate changes upon dissolution. For this experiment **1** (20 mg, 14.5 µmol) were dissolved in MeCN (1 ml) and subsequently evaporated in ambient conditions (*ca.* 1 h).

6. Small-angle X-ray diffraction

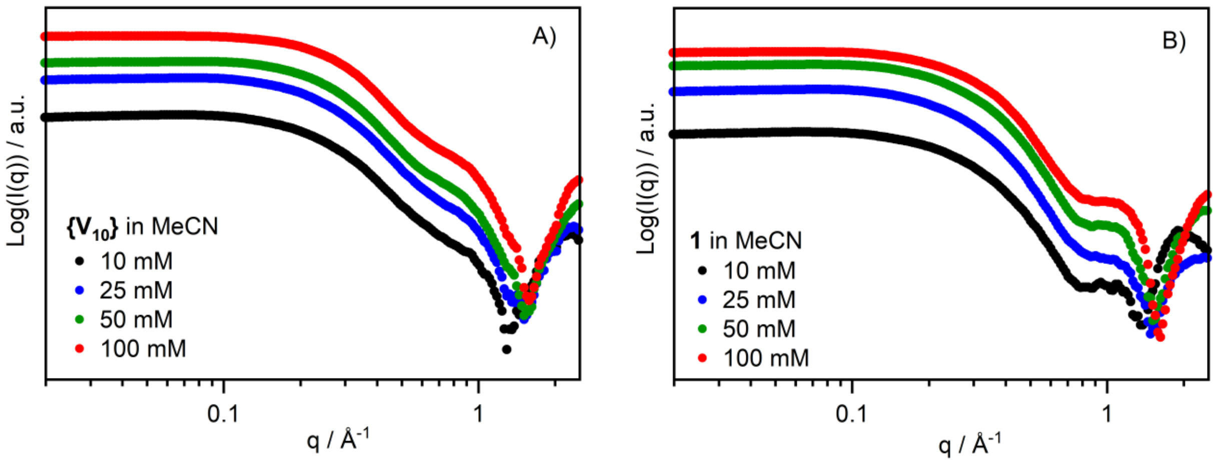

Figure S3 SAXS of $(n\mathrm{Bu}_4)_3[\mathrm{H}_3\mathrm{V}_{10}\mathrm{O}_{28}]$ (A) and **1** (B) for four different concentrations, dissolved in acetonitrile.

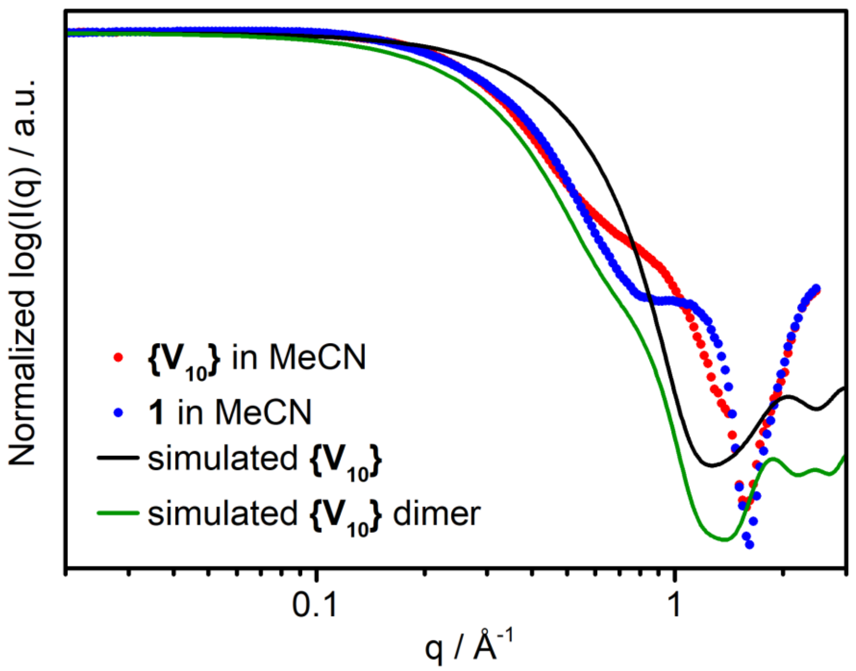

Figure S4 A) Experimental and simulated scattering curves, intensity normalized to $I(q)_{max}$ for ease of comparison. Experimental scattering curves are from 100 mM solutions.

7. X-ray photoelectron spectroscopy

Figure S5 shows XPS measurements and fitting of **1**, **{V₁₈}** and **{Ca₂V₁₈}**. The spectra were fitted with respect to the main O1s peak at 530.0 eV with O1s-$V^{5+}2p_{3/2}$ distance of 12.8 eV and O1s-$V^{4+}2p_{3/2}$ distance of 14.2 eV. The V $2p_{3/2}$ – V $2p_{1/2}$ splitting was set to 7.3 eV.[11–13] Additionally, a $V^{5+}2p_{3/2}$ satellite was fitted at $V^{5+}2p_{3/2}$+13.7 eV.[11] Comparison of the integrated peak intensity yielded V^{IV}:V^{V} ratios of 6.8:3.2 (**1**), 15.9:2.1 (**{V_{18}}**) and 17.0:0.9 (**{Ca_2V_{18}}**).

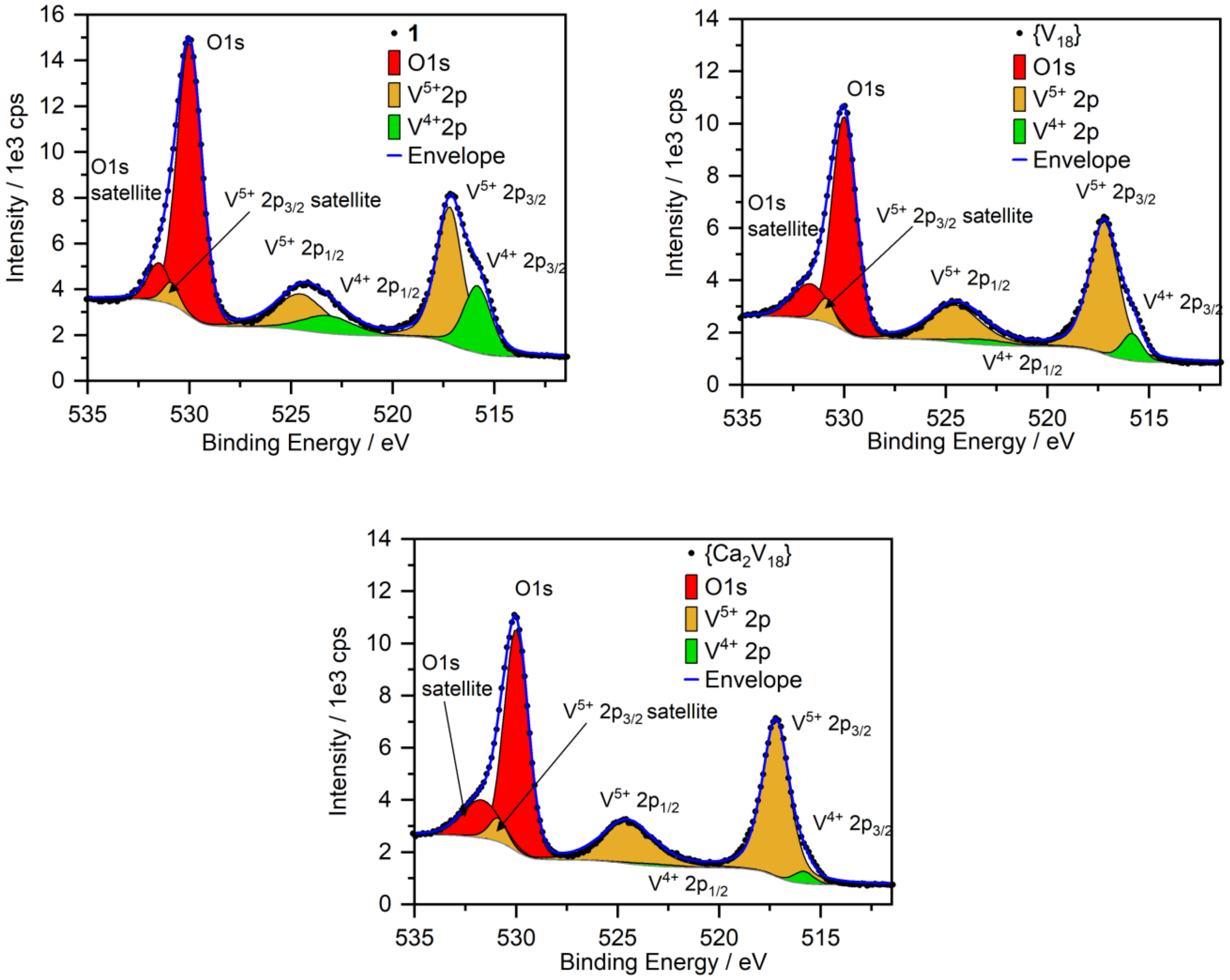

Figure S5 XPS spectrum of **1** (top left), **{V_{18}}** (top right) and **{Ca_2V_{18}}** (bottom) in the O1s/V2p area.

8. Thermogravimetric Analysis

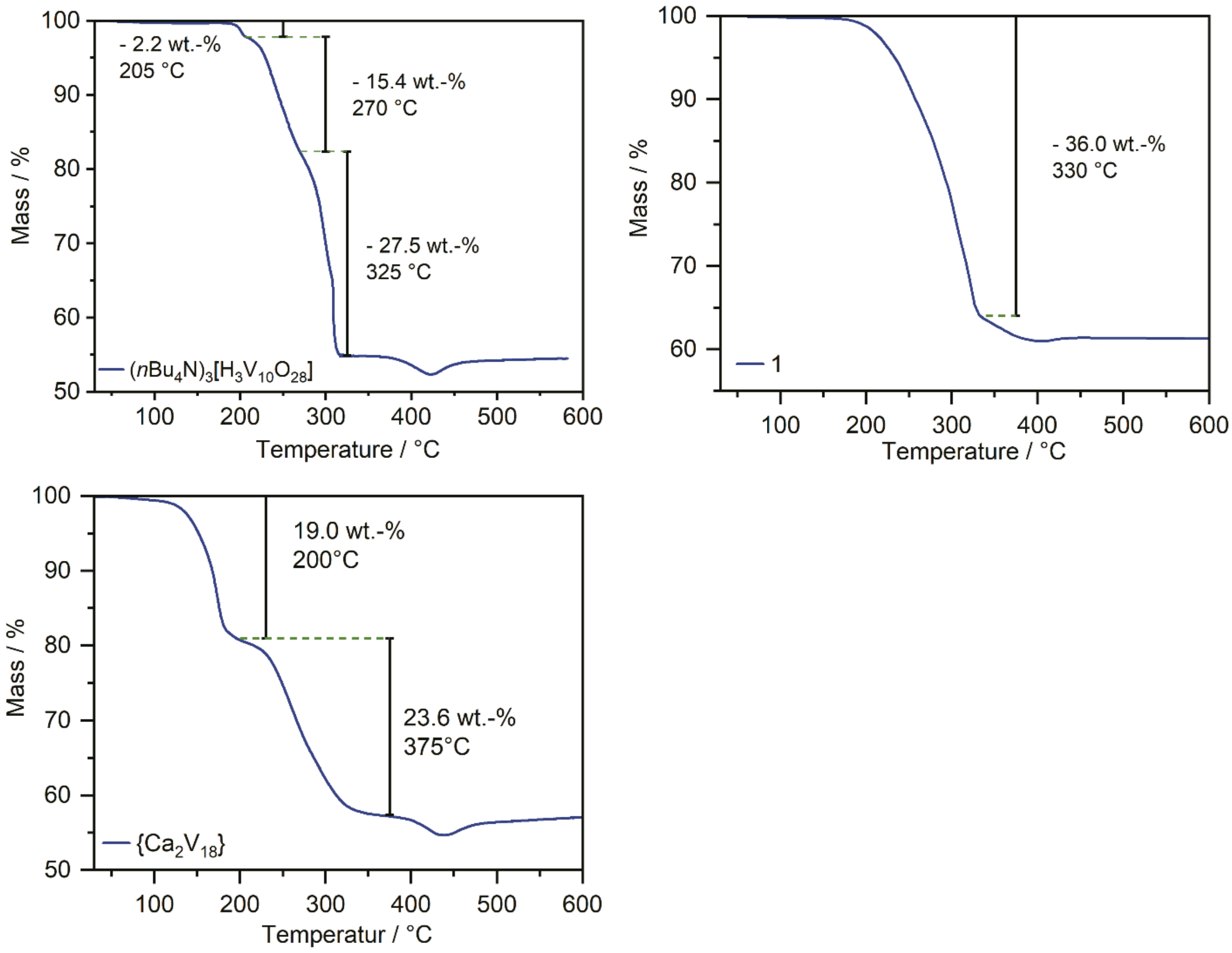

Figure S6 Top left: Thermogravimetric analysis (under air) of **{V$_{10}$}** showing a weight loss corresponding to one acetonitrile molecule until 205 °C (obs.: 2.2 wt.-%; calcd.: 2.4 wt.-%) followed by three (nBu$_4$N)$^+$ molecules until 325 °C (obs.: 42.9 wt.-%; calcd.: 42.0 wt.-%). A shoulder around 270 °C indicates the loss of one (nBu$_4$N)$^+$ molecule (obs.: 15.4 wt.-%; calcd.: 14.0 wt.-%) before the other two. **Top right**: Thermogravimetric analysis (under air) of **1** showing a weight loss corresponding to two (nBu$_4$N)$^+$ molecules until 330 °C (obs.: 36.0 wt.-%; calcd.: 35.2 wt.-%). **Bottom left**: TGA of **{Ca$_2$V$_{18}$}** showing a weight loss corresponding to one acetonitrile molecule and six ethyl acetate molecules until 200 °C (obs.: 19.0 wt.-%; calcd.: 19.2 wt.-%) and subsequent loss of 3 (nBu$_4$N)$^+$ molecules until 375 °C (obs.: 23.6 wt.-%; calcd.: 23.3 wt.-%).

9. X-ray Absorption spectroscopy (XAS)

a. X-ray absorption near-edge spectroscopy (XANES)

The near-edge structure of **1** resembles that of the precursor $(n\text{Bu}_4\text{N})_3[\text{H}_3\text{V}_{10}\text{O}_{28}]$, suggesting a close structural similarity between both compounds as proposed in the manuscript. A slight shift of the pre-edge feature around 5470 eV is observed for **1** (see Fig. S5 inset) in comparison to the decavanadate. The intensity of the pre-edge feature increases. This has been observed for similar systems in literature and was assigned to a loss of symmetry and a possible decrease in vanadium coordination.[14]

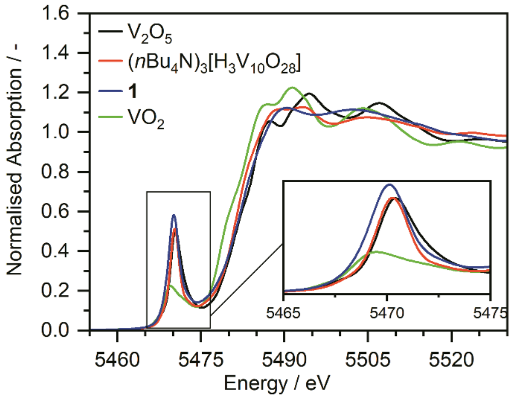

Figure S7 Plot of the V K-edge XANES data comparing $(n\text{Bu}_4\text{N})_3[\text{H}_3\text{V}_{10}\text{O}_{28}]$ and **1** with $\text{V}^{\text{V}}{}_2\text{O}_5$ and $\text{V}^{\text{IV}}\text{O}_2$ references. **Inset**: Enlargement of the pre-edge features around 5470 eV.

b. Extended X-ray absorption fine-structure spectroscopy (EXAFS)

To gain further insight on the coordination sphere of vanadium in **1**, EXAFS was performed and fitted based on the crystal structure of $(nBu_4N)_3[H_3V_{10}O_{28}]$ (CCDC: 159374).[15] The decavanadate cluster displays three different V atoms V_A (orange), V_B (yellow) and V_C (brown) and four different oxygen atoms (O_t, $O_{\mu2}$, $O_{\mu3}$, O_c) (see Fig. S6). This results in different V-O bonds with increasing bond length V-O_t (purple), V-$O_{\mu2}$ (grey), V-$O_{\mu3}$ (green) and V-O_c (blue). The results of the fits based on this model for $(nBu_4N)_3[H_3V_{10}O_{28}]$ and **1** can be found in Table S3 and S4.

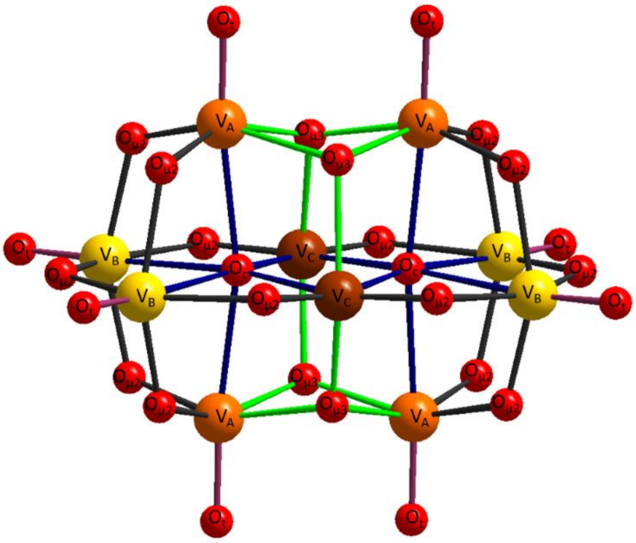

Figure S 8 Ball-and-stick representation of the decavanadate cluster showing the different bonds between vanadium and oxygen.

Table S3 Results of the V K-edge EXAFS fit for $(nBu_4N)_3[H_3V_{10}O_{28}]$. For the fit, a k-range of 3 - 11.2 and an R-range of 0.8 - 3.4 was chosen. χ^2 = 2019; reduced χ^2 = 859; R-factor 0.005. The passive electron reduction factor S_0^2 = 0.9 and the edge energy shift E_0 = -1.8 eV were optimized to be the same value for all listed scattering paths. The mean square relative displacement parameter (σ^2) for V-O and for V-V interactions were restrained to be the same, respectively. Coordination numbers were derived from the crystal structure and grouped by bond length.

Parameter	Coordination number	σ^2	R
**V-O_1**	1.2	0.007 ± 0.002	1.61 ± 0.03
**V-O_2**	1.8	0.007 ± 0.002	1.72 ± 0.03
**V-O_3**	2.2	0.007 ± 0.002	1.94 ± 0.03
**V-O_4**	0.8	0.007 ± 0.002	2.60 ± 0.04
**V-V_1**	3.2	0.005 ± 0.002	3.06 ± 0.02
**V-V_2**	0.8	0.005 ± 0.002	2.84 ± 0.02
**V-V_3**	0.8	0.005 ± 0.002	3.21 ± 0.02

Table S4 Results of the V K-edge EXAFS fit for 1. For the fit, a k range of 3 - 11.2 and an R range of 0.8 - 3.4 was chosen. χ^2 = 460; reduced χ^2 = 137; R-factor 0.002. The passive electron reduction factor S_0^2 = 0.9 and the edge energy shift E_0 = 0.1 eV were optimized to the same value for all listed scattering paths. The mean square relative displacement parameter (σ^2) for V-O and for V-V interactions were restrained to be the same, respectively. Coordination numbers were derived from the crystal structure of $(nBu_4N)_3[H_3V_{10}O_{28}]$. The coordination numbers were varied to improve the overall fit and account for the changes during heat treatment.

Parameter	Coordination number	σ^2	R
**V-O_1**	1.5	0.003 ± 0.002	1.59 ± 0.03
**V-O_2**	2.1	0.003 ± 0.002	1.78 ± 0.03
**V-O_3**	2.4	0.003 ± 0.002	1.95 ± 0.03
**V-V_1**	1.8	0.012 ± 0.004	3.01 ± 0.02
**V-V_3**	0.8	0.012 ± 0.004	3.29 ± 0.02

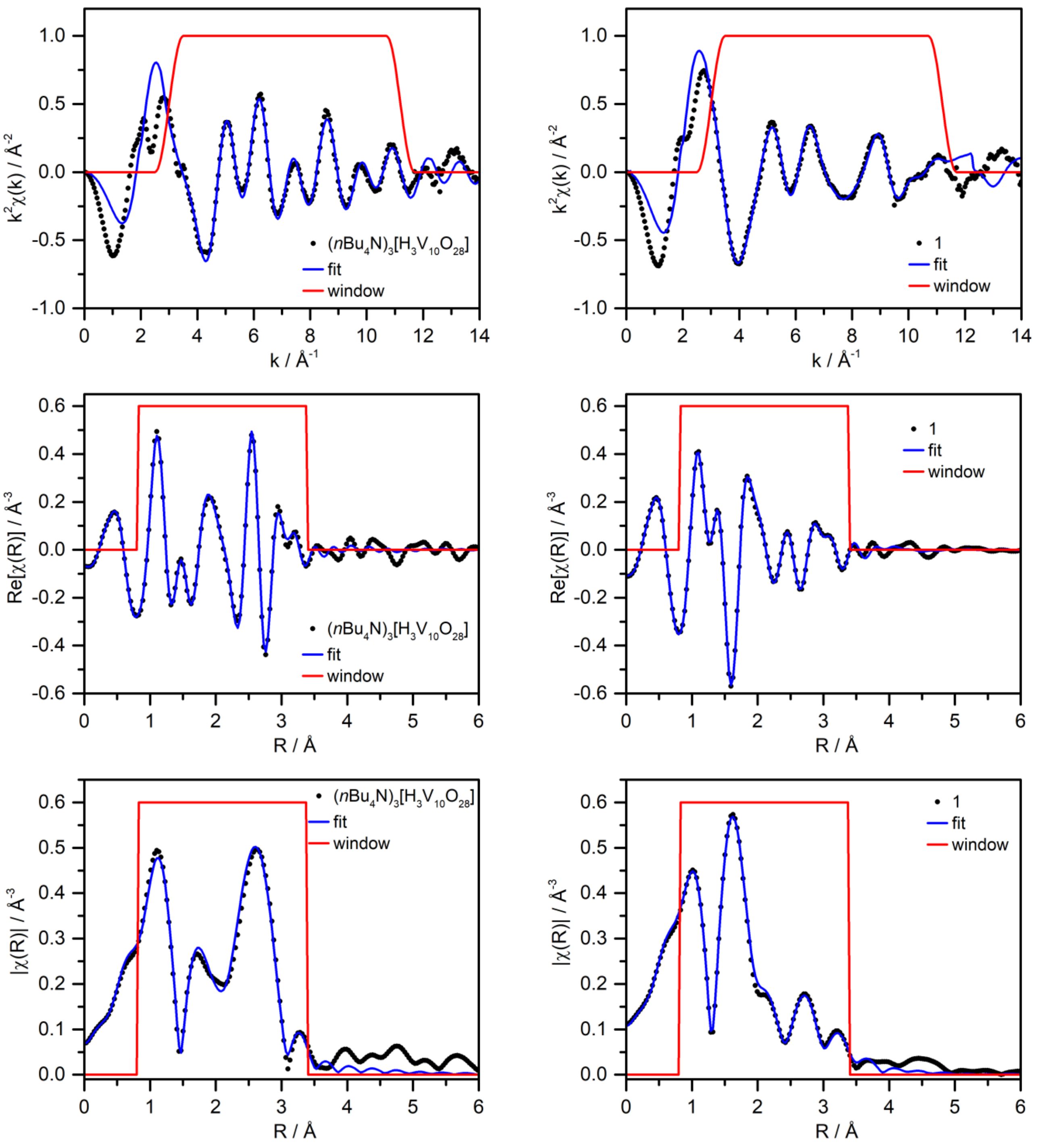

Figure S9 Top left: Fitting of $k^2\chi(k)$ of (nBu$_4$)$_3$[$H_3V_{10}O_{28}$] in the k-space. **Middle left**: Fitting of the real part of the Fourier transformed EXAFS Re[χ(R)] of (nBu$_4$)$_3$[$H_3V_{10}O_{28}$]. **Bottom left**: Fitting of the magnitude of the Fourier-transformed EXAFS |χ(R)| of (nBu$_4$)$_3$[$H_3V_{10}O_{28}$]. **Top right**: Fitting of $k^2\chi(k)$ of **1** in the k-space. **Middle right**: Fitting of the real part of the Fourier transformed EXAFS Re[χ(R)] of **1**. **Bottom right**: Fitting of the magnitude of the Fourier-transformed EXAFS |χ(R)| of **1**.

10. MAS-^{51}V-NMR spectroscopy

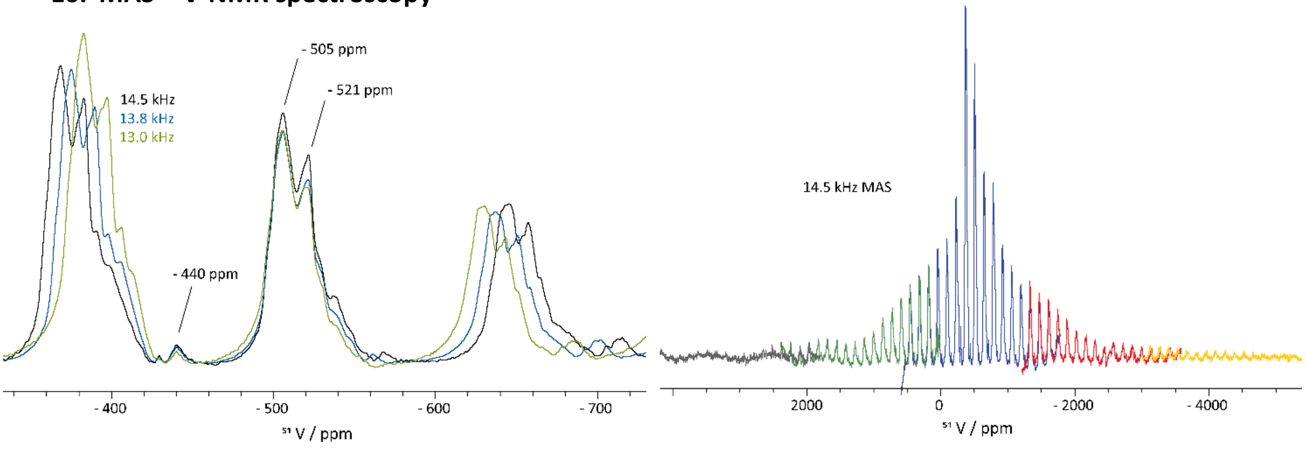

Figure S10 Left: Extract of the ^{51}V solid-state NMR spectrum of **{V_{10}}** between -330 and -730 ppm recorded at three different MAS frequencies. **Right**: Full ^{51}V NMR spectrum of **{V_{10}}** between 4000 and -4500 ppm measured at 14.5 kHz MAS. Due to the width of the spinning sideband pattern, several spectra with different offsets were recorded individually and plotted together as indicated by different colors.

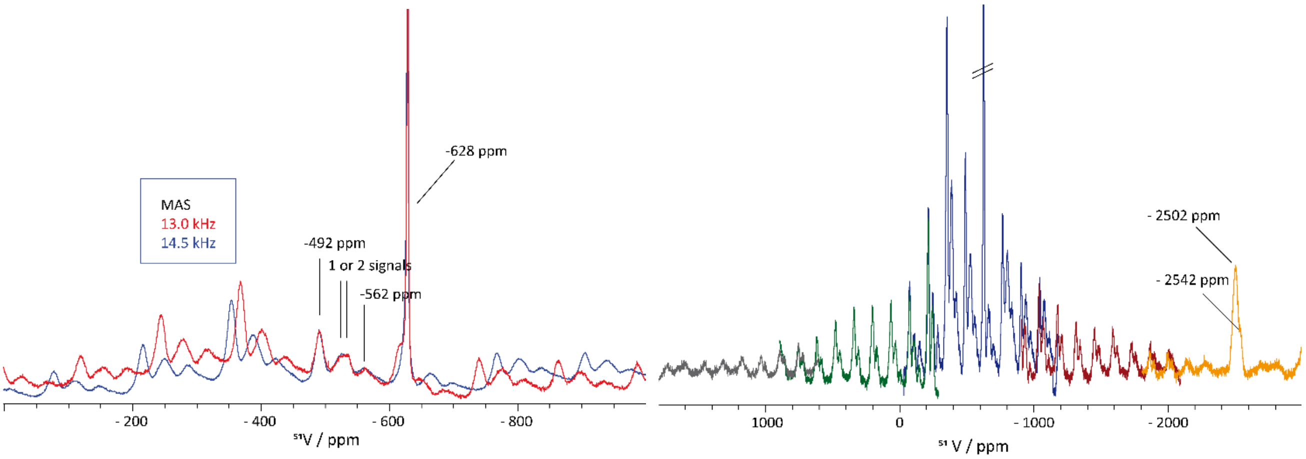

Figure S11 Left: Extract from the ^{51}V solid-state NMR spectrum of **1** between -100 and -1000 ppm recorded at two different MAS frequencies. **Right**: Full ^{51}V NMR spectrum between 1800 and -3000 ppm. Due to the width of the spinning sideband pattern several spectra with different offset and a spectral width of 1000 ppm were recorded and plotted together (indicated by different colors). The signal at -2500 ppm is most likely caused by the radio frequency coil of the probe (^{63}Cu).[16]

To assess the effect of reduced vanadium centers in close proximity, MAS-^{51}V-NMR of the literature-known $(n\mathrm{Bu_4N})_3[(\mathrm{Me_2NH_2})_2\mathrm{V^V}_{12}\mathrm{O_{32}Cl}]$[17] and $(n\mathrm{Bu_4N})_2[\mathrm{Ca_2(C_3H_7NO)_3V^{IV}V^V}_{11}\mathrm{O_{32}Cl}]$[18] were compared and a shift of the main signals to more negative ppm values was observed for the reduced species.

MAS-^{51}V-NMR of $(n\mathrm{Bu_4N})_3[(\mathrm{Me_2NH_2})_2\mathrm{V^V}_{12}\mathrm{O_{32}Cl}]$ / ppm: -552 to -555, -577.

MAS-^{51}V-NMR of $(n\mathrm{Bu_4N})_2[\mathrm{Ca_2(C_3H_7NO)_3V^{IV}V^V}_{11}\mathrm{O_{32}Cl}]$ / ppm: -563, -586.

11. Electrochemistry of {V_{18}}

CV of **{V_{18}}** in the range between -1.3 – 1.0 V shows four quasi-reversible redox couples at $E_{I/I'}$ = 0.67 V, $E_{II/II'}$ = 0.33 V, $E_{III/III'}$ = -0.43 V, $E_{IV/IV'}$ = -0.79 V and one more less reversible process until -1.6 V at $E_{V/V'}$ = -1.34 V (all vs. Fc^+/Fc).

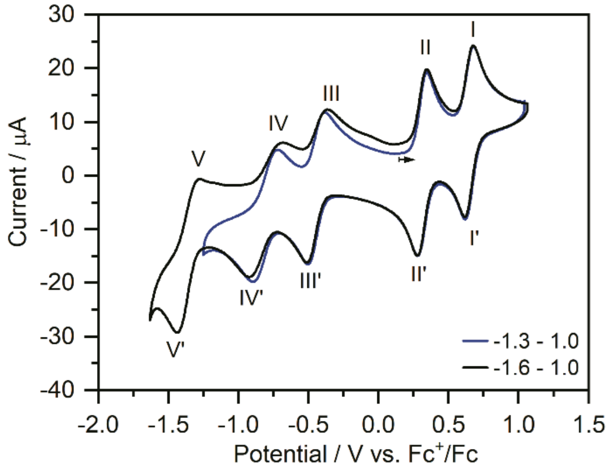

Figure S12 Cyclic voltammogram of **{V_{18}}** between -1.3 – 1.0 V (blue) and -1.6 – 1.0 V (black) in de-aerated, anhydrous acetonitrile containing 0.1 M (nBu$_4$N)PF$_6$.

12. Mass spectrometry

c. HR-ESI-MS of 1

Table S5 Vanadium oxide species observed in negative ion mode HR-ESI mass spectroscopy of **1**.

Observed m/z	Calculated m/z	Charge	Assignment	Known cluster (c), fragments (f) or “virtual cluster” (v)
1508.8426	1508.8037	2-	$[V^{IV}_{32}V^{V}_{4}O_{74}]^{2-}$	V
1409.9046	1409.8770	2-	$(nBu_4N)_2[V^{IV}_{2}V^{V}_{8}O_{26}]^{2-}$	C
1250.5523	1250.5574	2-	$[H_3V^{IV}_{11}V^{V}_{17}O_{67}]^{2-}$	V
1239.2858	1239.3029	2-	$(nBu_4N)_3[H_3V^{IV}_{8}V^{V}_{10}O_{52}]^{2-}$	V
826.3870	826.3793	1-	$[V^{V}_{9}O_{23}]^{-}$	F
644.5212	644.5162	1-	$[V^{V}_{7}O_{18}]^{-}$	F
553.5904	553.5855	2-	$[V^{V}_{12}O_{31}]^{2-}$	F
504.1250	504.1212	2-	$[V^{IV}V^{V}_{10}O_{28}]^{2-}$	F
462.6575	462.6543	1-	$[V^{V}_{5}O_{13}]^{-}$	F

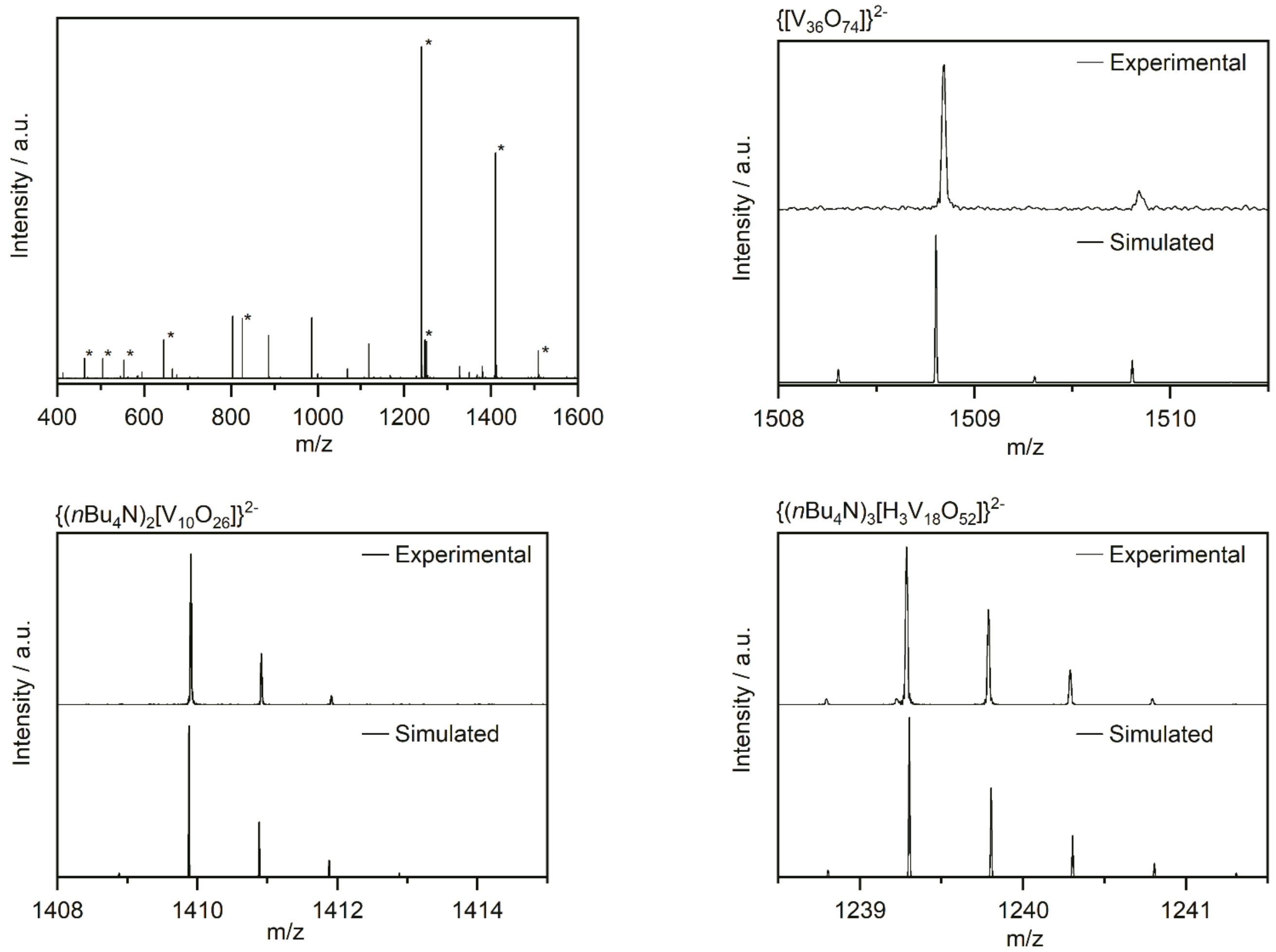

Figure S13 Top left: High-resolution electrospray ionization mass-spectrometry of **1** (ca. $5x10^{-5}$ M in acetonitrile). **Top right:** Experimental and simulated ESI mass spectrum of the biggest fragment $[V^{V}_{4}V^{IV}_{32}O_{74}]^{2-}$ observed at m/z = 1508.8426. **Bottom left:** Experimental and simulated ESI mass spectrum of $(nBu_4N)_2[V^{V}_{2}V^{IV}_{8}O_{26}]^{2-}$ observed at m/z = 1409.9046. **Bottom right:** Experimental and simulated ESI mass spectrum of $(nBu_4N)_3[H_3V^{V}_{10}V^{IV}_{8}O_{52}]^{2-}$ observed at m/z = 1239.2858.

d. HR-ESI-MS of {Ca_2V_{18}}

Table S 6 Calcium vanadate species observed in negative ion mode HR-ESI mass spectrometry of **{Ca_2V_{18}}**.

Observed m/z	Calculated m/z	Charge	Assignment
2311.2915	2311.2303	1-	$\{(n\mathrm{Bu_4N})_2[\mathrm{Ca_2V^{V}_{18}O_{48}(NO_3)}]\}^{1-}$
1672.8928	1672.8517	4-	$\{(n\mathrm{Bu_4N})_5[\mathrm{Ca_2V^{V}_{18}O_{48}(NO_3)}]_3\}^{4-}$
1540.8594	1540.8204	3-	$\{(n\mathrm{Bu_4N})_4[\mathrm{Ca_2V^{IV}V^{V}_{17}O_{48}(NO_3)}][\mathrm{Ca_2V^{V}_{18}O_{48}(NO_3)}]\}^{3-}$
1460.0801	1460.0588	3-	$\{(n\mathrm{Bu_4N})_3[\mathrm{Ca_2V^{V}_{18}O_{48}(NO_3)}]_2\}^{3-}$
1155.6335	1155.6154	2-	$\{(n\mathrm{Bu_4N})_2[\mathrm{Ca_2V^{IV}V^{V}_{17}O_{48}(NO_3)}]\}^{2-}$
1034.4827	1034.4731	2-	$\{(n\mathrm{Bu_4N})[\mathrm{Ca_2V^{V}_{18}O_{48}(NO_3)}]\}^{2-}$
1003.4909	1003.4911	2-	$\{(n\mathrm{Bu_4N})\mathrm{H_2}[\mathrm{Ca_2V^{IV}_{10}V^{V}_{8}O_{44}(NO_3)}]\}^{2-}$
882.3443	882.3487	2-	$\{\mathrm{H_2}[\mathrm{Ca_2V^{IV}_{9}V^{V}_{9}O_{44}(NO_3)}]\}^{2-}$
608.8925	608.8873	3-	$[\mathrm{Ca_2V^{V}_{18}O_{48}(NO_3)}]^{3-}$
588.2304	588.2326	3-	$\{\mathrm{H_2}[\mathrm{Ca_2V^{IV}_{10}V^{V}_{8}O_{44}(NO_3)}]\}^{3-}$
344.8552	344.8247	6-	$[\{(n\mathrm{Bu_4N})[\mathrm{Ca_2V^{IV}_{4}V^{V}_{14}O_{48}(NO_3)}]\}^{6-}$

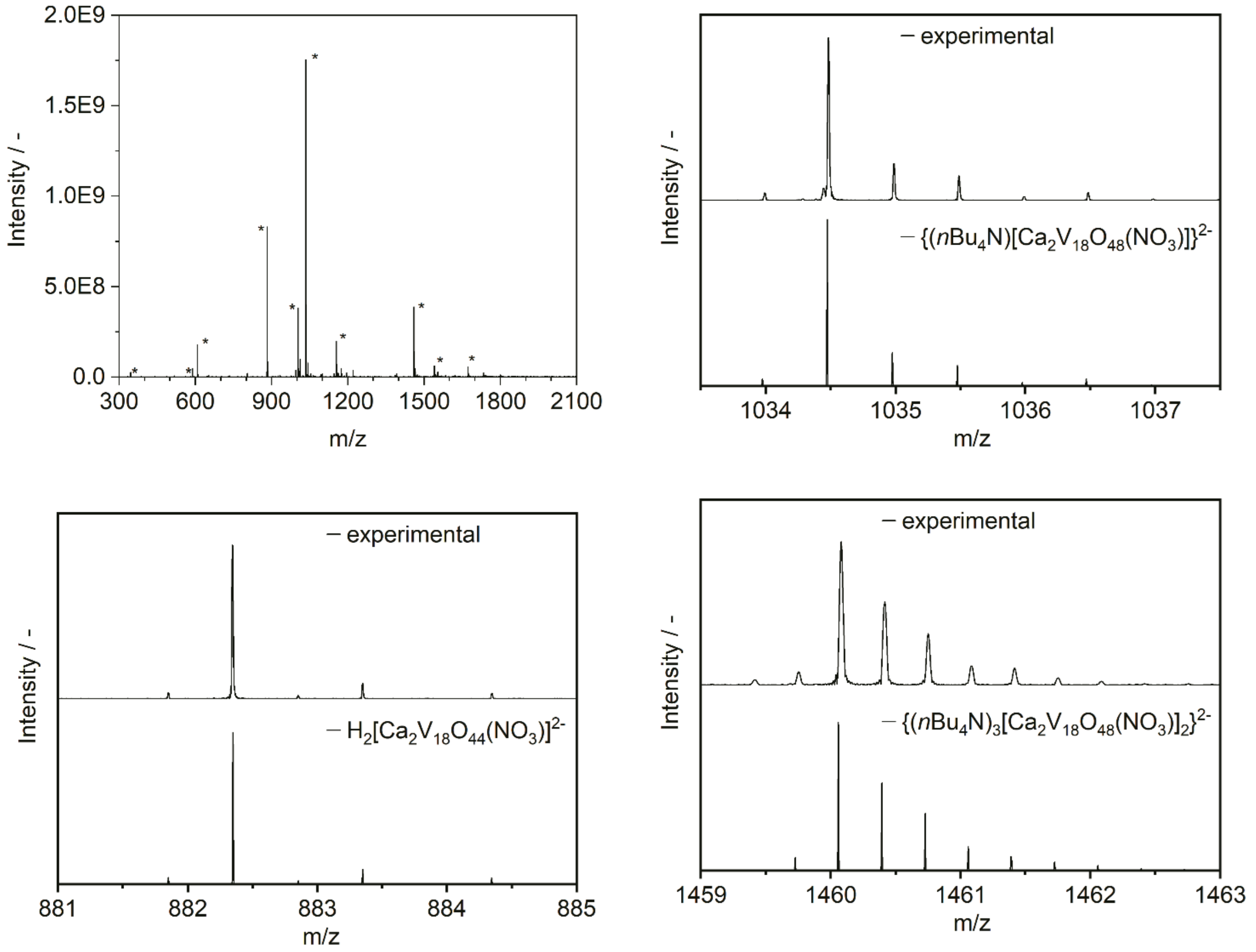

Figure S14 Top left: High-resolution electrospray ionization mass-spectrometry of **{Ca_2V_{18}}** (ca. 5×10^{-5} M in acetonitrile). **Top right:** Experimental and simulated ESI mass spectrum of the full cluster (main signal) $\{(n\mathrm{Bu_4N})[\mathrm{Ca_2V^{V}_{18}O_{48}(NO_3)}]\}^{2-}$ observed at m/z = 1034.4827. **Bottom left:** Experimental and simulated ESI mass spectrum of the anion $\{\mathrm{H_2}[\mathrm{Ca_2V^{IV}_{9}V^{V}_{9}O_{44}(NO_3)}]^{2-}$, which could be a decomposition product, observed at m/z = 882.3443. **Bottom right:** Experimental and simulated ESI mass spectrum of the dimer fragment $\{(n\mathrm{Bu_4N})_3[\mathrm{Ca_2V^{V}_{18}O_{48}(NO_3)}]_2\}^{2-}$ observed at m/z = 1460.0801.

13. References

[1] X. Zuo, G. Cui, K. M. Merz, L. Zhang, F. D. Lewis, D. M. Tiede, *Proc. Natl. Acad. Sci.* **2006**, *103*, 3534–3539.

[2] A. H. Clark, J. Imbao, R. Frahm, M. Nachtegaal, *J. Synchrotron Radiat.* **2020**, *27*, 551–557.

[3] B. Ravel, M. Newville, *J. Synchrotron Radiat.* **2005**, *12*, 537–541.

[4] J. J. Rehr, R. C. Albers, S. I. Zabinsky, *Phys. Rev. Lett.* **1992**, *69*, 3397–3400.

[5] Walter G. Klemperer, in *Inorg. Synth. Vol. 27*, John Wiley & Sons, **1990**, p. 83.

[6] George M. Sheldrick, *Acta Crystallogr. Sect. C* **2015**, *71*, 3–8.

[7] O. V. Dolomanov, L. J. Bourhis, R. J. Gildea, J. A. K. Howard, H. Puschmann, *J. Appl. Crystallogr.* **2009**, *42*, 339–341.

[8] B. Y. R. H. Blessing, *Acta Crystallogr. Sect. A* **1995**, *51*, 33–38.

[9] J. Forster, B. Rösner, R. H. Fink, L. C. Nye, I. Ivanovic-Burmazovic, K. Kastner, J. Tucher, C. Streb, *Chem. Sci.* **2013**, *4*, 418–424.

[10] Y. Koyama, Y. Hayashi, K. Isobe, *Chem. Lett.* **2008**, *37*, 578–579.

[11] G. Silversmit, D. Depla, H. Poelman, G. B. Marin, R. De Gryse, *J. Electron Spectros. Relat. Phenomena* **2004**, *135*, 167–175.

[12] E. Hryha, E. Rutqvist, L. Nyborg, *Surf. Interface Anal.* **2012**, *44*, 1022–1025.

[13] M. Demeter, M. Neumann, W. Reichelt, *Surf. Sci.* **2000**, *454–456*, 41–44.

[14] M. P. M. Marques, D. Gianolio, S. Ramos, L. A. E. Batista de Carvalho, M. Aureliano, *Inorg. Chem.* **2017**, *56*, 10893–10903.

[15] S. Nakamura, T. Ozeki, *J. Chem. Soc. Dalt. Trans.* **2001**, 472–480.

[16] K. Ooms, T. Polenova, A.-M. Shough, D. J. Doren, M. J. Nash, R. F. Lobo, *J. Phys. Chem. C* **2009**, *113*, 10477–10484.

[17] K. Kastner, J. T. Margraf, T. Clark, C. Streb, *Chem. - A Eur. J.* **2014**, *20*, 12269–12273.

[18] S. Greiner, B. Schwarz, M. R. Ringenberg, M. Dürr, I. Ivanovic-Burmazovic, M. Fichtner, M. Anjass, C. Streb, *Chem. Sci.* **2020**, *11*, 4450–4455.

14. Author Contributions

A.L., K.B., J.H., M.A. and S.G. performed synthesis and characterization. K.B., M.A., J.H and S.G. performed electrochemistry. S.G. performed scXRD measurement and analysis. A.H.C. performed XAS measurements and A.H.C. and S.G. analyzed the data. A.C.P. performed MAS-NMR spectroscopy. J.B. and M.N. performed SAXS. S.G., M.A. and C.S. conceived the experiments and wrote the manuscript.
